# An extended Arctic proxy temperature database for the past 2,000 years

**DOI:** 10.1038/sdata.2014.26

**Published:** 2014-08-19

**Authors:** Nicholas P. McKay, Darrell S. Kaufman

**Affiliations:** 1 School of Earth Sciences and Environmental Sustainability, Northern Arizona University, Flagstaff, Arizona 86011, USA

## Abstract

Robust climate reconstructions of the most recent centuries and millennia are invaluable for placing modern warming in the context of natural variability. Here we present an extended and revised database (version 1.1) of proxy temperature records recently used to reconstruct Arctic temperatures for the past 2,000 years. The datasets are presented in a machine-readable format, and have been extended with the geochronologic data and consistently generated time-uncertain ensembles, which will be useful in future analyses of the influence of geochronologic uncertainty. A standardized description of the seasonality of the temperature response for each record, as reported by the original authors, is also included to motivate a more nuanced approach to integrating records with variable seasonal sensitivities. Despite the predominance of seasonal, rather than annual, temperature responders in the database, comparisons with the instrumental record of temperature suggest that, as a whole, the datasets best record annual temperature variability across the Arctic, especially in northeast Canada and Greenland, where the density of records is highest.

## Background & Summary

An accurate understanding of the past one to two thousand years of Earth's climate history is critical for placing recent warming in the context natural climate variability. Consequently, extensive efforts have been made to reconstruct regional^1^, hemispheric^2–4,2–4,2–4^, and global-scale temperature changes^3,5^ over the most recent centuries and millennia. Predominantly, the evidence used to inform these reconstructions has been derived from tree-ring records, because they are annually resolved, precisely dated, and geographically widespread, especially in the mid-latitudes of the Northern Hemisphere. Increasingly, efforts have been made to incorporate paleoclimate evidence from other sources, such as lake and marine sediments, and records from glacial ice, and cave speleothems, primarily to expand the geographic and temporal coverage of the reconstructions. This is particularly true for the Arctic, where tree-ring records are more scarce, and where extensive paleoclimate research has taken advantage of the widespread presence of proxy climate records from lake sediment and glacial ice. This has led to a long history of multiproxy climate reconstructions for the late Holocene in the Arctic^1,6,7^. Incorporating these diverse data also brings additional challenges, largely due to how they differ from tree-ring records. Specifically, records derived from sediment, ice, and cave calcite contain varying degrees of chronological uncertainty, are commonly non-annually resolved and unevenly spaced, and each filter climate in different ways. These characteristics typically invalidate the assumptions underlying most statistical climate reconstruction techniques^8^; however, efforts to accommodate these data types^9^ and to assess the influence of chronological uncertainty^10^ are beginning to emerge.

Here we present an Arctic proxy temperature database for the past 2,000 years. The database is a revised version of the one used to reconstruct temperature in the Arctic for the past 2,000 years, which was recently included as part of the global summary by the Past Global Changes (PAGES) 2k Consortium^1^. In addition, we expanded the database by including consistently determined chronological uncertainty estimates for every record, except tree-ring records. These data are needed to quantify the influence of age uncertainty in climate reconstructions, but are rarely accessible to researchers aiming to develop large-scale climate reconstructions. This database also complements the recent Arctic Holocene Transitions (AHT) database^11^, a well-formatted collection of Arctic paleoclimate records for the Holocene. The overlap between the two datasets is minimal (9% of the sites in the AHT database are also included in this collection) because the AHT database includes records that extend further back at lower resolution; all records go back to at least 6000 years ago, and most extend 9000 years. Additionally, the AHT database only includes the geochronology data for radiometrically dated records, and does not include age ensembles for addressing age uncertainties. To our knowledge, the collection presented in this data descriptor is the first compilation of proxy climate data to include age ensembles, or age uncertainty estimates of any kind for layer-counted records.

## Methods

### Data aggregation and formatting

**Figure 1:**
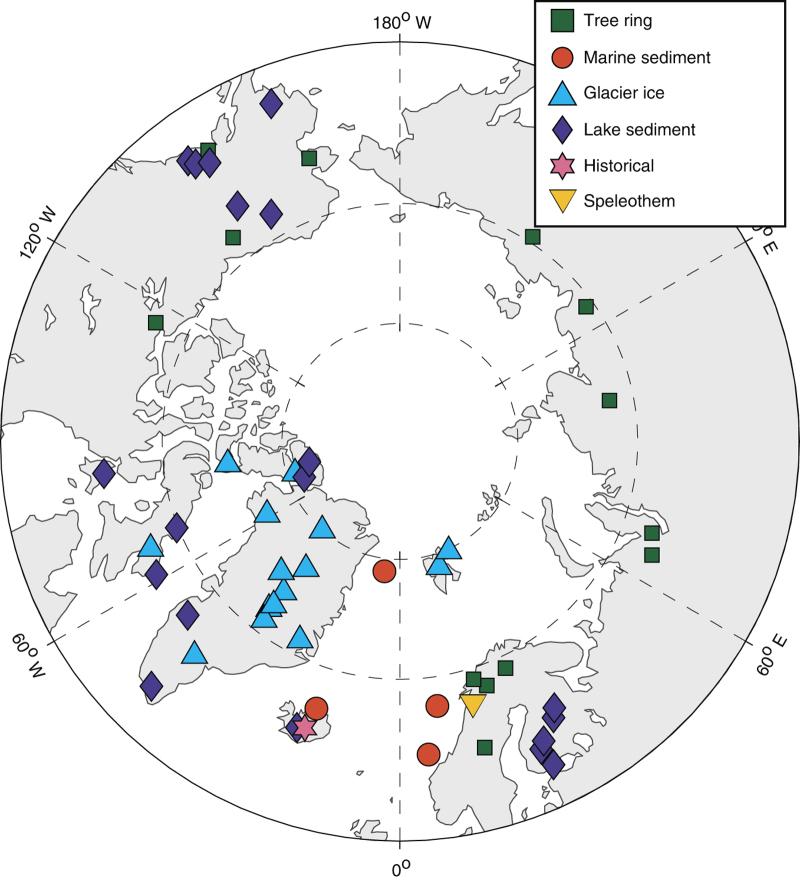
Polar projection showing the location and archive type of proxy temperature records in the PAGES Arctic 2k database. Information about each site is listed in Table 1.

The database presented here is a revised version of the one used for the Arctic region of the PAGES 2k Network^1^ (Figure 1). Each revision is described below and in Table 1. The records selected were required to meet several criteria. Specifically all records:

1. are from north of 60°N;
2. extend back in time to at least 1500 AD;
3. have an average sample resolution less than 50 years;
4. have at least one age control point every 500 years;
5. have been published in a peer-reviewed journal, where evidence is presented documenting that the record is sensitive to temperature. This evidence may be statistical (e.g., correlation with nearby instrumental temperature data), or mechanistic (e.g., description by the authors of mechanisms by which the archive senses temperature change).

In several cases, the fifth criterion above is not met throughout the entire record (e.g., following AD 1720, agriculture nearby Lake Korttajärvi disrupts the temperature sensitivity of the record^12^). In these cases, we excluded the section of the record that violates this criterion.

### Geochronology

In this study, we substantially expand the PAGES Arctic 2k database by including formatted geochronology data (e.g., radiocarbon ages and associated data) for the radiometrically-dated records, and systematically determined age-ensembles for all of the radiometrically-dated and layer-counted records in the database.

### Radiometrically-dated records

For each radiometrically-dated record, we developed a new age-depth model using the original geochronology data from each site and the Bayesian ACcumulatiON (BACON) algorithm^13^. BACON is a Bayesian age-modeling routine written for the software package R that takes advantage of prior knowledge about the distribution and autocorrelation structure of sedimentation rates in a sequence. The algorithm employs an adaptive Markov Chain Monte Carlo algorithm that allows for Bayesian learning to update the sedimentation-rate distribution.

The new age models do not replace those of the original study. Indeed, it is likely that the original investigators incorporated expert knowledge into the development of the original age models that we cannot replicate. Although the revised best-estimate age models may, in some cases, be inferior, there are two advantages to our approach. First, by systematically determining ages using a consistent methodology, we eliminate the aspect of age uncertainty and bias when comparing two records due to choices made during age modelling and the nuances of the many approaches originally used. Second, for each site, we extract a subset of age-ensemble members, which will facilitate future efforts to quantify the influence of age uncertainty in Arctic mean and temperature field reconstructions. This is important because nearly all of the original age models did not consider age-uncertain ensembles, and the data are not available for the few that did.

### Layer-counted records

The PAGES Arctic 2k database includes 26 records from annually banded (varved) lake sediment and glacier ice for which the chronologies are developed by layer counting. The timeline for tree-ring records are also based on layer counting for which cross-dating among many samples makes tree-ring chronologies robust with negligible error^14,15^. Age uncertainty for annually banded sediments and ice cores typically increases with age. Although such records can often reach subannual precision, replication is more difficult and costly than with tree ring records, and consequently, cross-dating is rare, but possible with sufficient replication.

To develop time-uncertain ensembles for the layer-counted records, we used BAM (Banded Age Model), a probabilistic model of age errors in layer-counted chronologies^16^. The model allows a flexible parametric representation of such errors (either as Poisson or Bernoulli processes), and separately considers the possibility of double counting or missing a band. For each layer-counted chronology, we used BAM with published over- and under-counting estimates from the original study of each record (Table 1). When such estimates were not available, we applied conservative estimates of 1% for both over- and under-counting.

### Arctic-wide temperature reconstruction

#### Changes from PAGES 2k Consortium (2013)

Here we present an Arctic regional temperature reconstruction that revises the one published recently by the PAGES 2k Consortium^1^. The revisions include updating records using more recent published studies from three sites^17–19,17–19,17–19^, and correcting several errors discovered following publication of the PAGES 2k Consortium article. Specifically:

1. Three records were removed because of insufficient evidence that they are sensitive to temperature^20–22,20–22,20–22^.
2. Sections of five records^23–27,23–27,23–27,23–27,23–27^ that were interpreted by the authors to violate criterion 5 were removed.
3. The interpreted temperature relation of the series from Hvítárvatn^28^ was corrected from positive to negative.
4. A 50-year offset in the ages of the record from Lone Spruce Pond^29^ was corrected.
5. The coordinates of the Copper River tree-ring reconstruction^24^ were corrected.

For this study, we did not add any new records to the database, or those that satisfy other criteria. We refer to this revised database as version 1.1. Additional records, including those sensitive to other aspects of the climate system (e.g., precipitation), will be included during the ongoing phase 2 of the PAGES 2k project. We suggest the next version of the database that includes additional records be designated as ‘version 2’.

#### Temperature reconstruction

The PAGES 2k Consortium^1^ used the Pairwise Comparison method (PaiCo^9^) to reconstruct the average Arctic mean-annual temperature for the past 2,000 years. PaiCo is a type of composite-plus-scale method^8^ that is unique because it does not require annually sampled data, nor the assumption that the proxy-temperature relation is linear (only monotonic). These features made it ideal for the Arctic 2k reconstruction. Here we use PaiCo to replicate the Arctic temperature reconstruction^1^, including the changes to the proxy database described above, to evaluate how the revisions influence the reconstruction.

**Figure 2:**
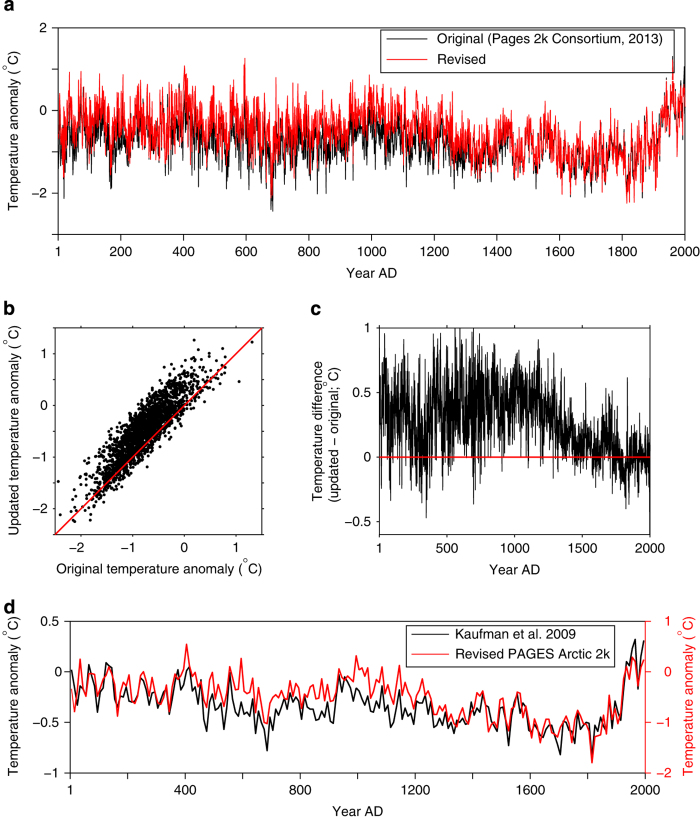
Effect of revising the PAGES Arctic 2k database on the Arctic annual temperature reconstruction published recently by the PAGES 2k Consortium^1^. (**a**) Reconstruction calculated using the original (black) and updated database presented here (red). (**b**) Scatter plot illustrating the influence of the revisions; 1:1 line shown in red. (**c**) Time-series of the differences in reconstructed temperature (revised—original); no change shown as red line. (**d**) Comparison between Kaufman *et al.* ^7^ Arctic—wide temperature reconstruction and the revised PAGES 2k Arctic reconstruction (averaged to decadal values). Note the factor-of-two difference in the temperature scales.

Overall, the database revisions have a fairly minor impact on the relative variability in the reconstruction, but they do affect the long-term trend (Figure 2). The primary change is a relative increase in reconstructed temperatures for most of the record, especially between AD 1–1300. This results in an amplified long-term cooling trend that preceded 20th century warming; 0.47 °C/kyr in the revised reconstruction compared to 0.29 °C/kyr in the original. Decadal—scale variability in the revised reconstruction is quite similar to that determined by Kaufman *et al.* ^7^; however, the variability is about twice as great in the revised PAGES Arctic 2k reconstruction (Figure 2d). This is likely due the averaging and scaling procedures used in the earlier study^7^.

## Data Records

The PAGES Arctic 2k database presented here (v 1.1) is archived at the National Oceanic and Atmospheric Administration's World Data Center for Paleoclimatology (WDC-Paleo) http://ncdc.noaa.gov/paleo/study/16973, and the data are formatted according to WDC-Paleo's most recent standards http://www.ncdc.noaa.gov/data-access/paleoclimatology-data/contributing. The database is also archived on figshare [Data Citation 1]. For each record, there are self-describing and machine-readable ascii-files that include extensive metadata (e.g., source, title, investigators, publications, site and chronology metadata, variable descriptions) as well as the time-series and chronology data (when appropriate). Additionally, each site (except tree-ring records) has a corresponding netCDF file that archives the age-model ensembles. These files include up to four large matrices, depending on archive type and resolution:

AgeYoungEns: An ensemble of age estimates corresponding to the upper extent of each sampled interval. Each column is a different ensemble member.

AgeOldEns: Same as AgeYoungEns, but for the lower extent of each sample.

BaconAgeEnsemble: Ensemble of age models determined by BACON^13^. Each column is a different ensemble member (radiometrically dated only).

BaconAgeEnsDepths: Depths corresponding to ages in BaconAgeEnsemble (radiometrically dated only).

AgeEns: An ensemble of age estimates for the annually-resolved, layer-counted records as determined by BAM^16^. Each column is a different ensemble member (layer counted only).

DataEns: An ensemble of time—series perturbed by the simulated age uncertainty in AgeEns. Each column is a different ensemble member (layer counted only).

The PAGES Arctic 2k temperature database includes records that infer past temperature variability from five types of natural archives. Each of these archives respond to temperature changes in different ways, and that signal is recorded in each archive's chemical, physical, or biological properties. An overview of the records comprising the database is presented in Table 1. A novel aspect of this collection is the specification of the seasonal correlation of each record as described in the original publication. As shown in Table 1, the seasonal response of the proxies is quite variable, yet most synthesis and reconstruction efforts, including both the original and revised reconstructions described above, disregard the potential for seasonal differences among records that bias inferred climate changes in the past. The first step towards a more realistic treatment of seasonality is a uniform handling of these metadata, and we hope that future compilations will make this a priority. Although the records are well-summarized in Table 1 and in each records file in the database, the full details behind the collection, analysis and interpretation of each of the 56 records in the database is beyond the scope of this compilation, and we refer readers to the original publications for that information^12,17-19,17-19,17-19,23-66,23-66,23-66,23-66,23-66,23-66,23-66,23-66,23-66,23-66,23-66,23-66,23-66,23-66,23-66,23-66,23-66,23-66,23-66,23-66,23-66,23-66,23-66,23-66,23-66,23-66,23-66,23-66,23-66,23-66,23-66,23-66,23-66,23-66,23-66,23-66,23-66,23-66,23-66,23-66,23-66,23-66,23-66,23-66^.

## Technical Validation

Evidence that the records in the database reflect past temperature variability can be found in the original publications associated with each record. Here, we examine the extent to which the database as a whole captures observed temperature variability in the region. To do this, we calculated field correlations and their significance between each record in the database and the Natiaonal Aeronautics and Space Administration's (NASA) Goddard Institute for Space Studies Surface Temperature Analysis (GISTEMP) product with 1,200-km smoothing^67^ during the period of overlap (AD 1880–2000). In this analysis, the time series for each site, as well as the Arctic-wide reconstruction, were correlated against the temperature record for every grid cell north of 60° N. Significance at each grid cell was determined using a Student's *T*-test following correction for autocorrelation^68^. All calculations were performed at the temporal resolution of the proxy values; annual-mean temperatures were used for the annually-sampled records, and averages of multiple years corresponding to the sampling of the low-resolution records were calculated to correlate with the lower-resolution records.

**Figure 3:**
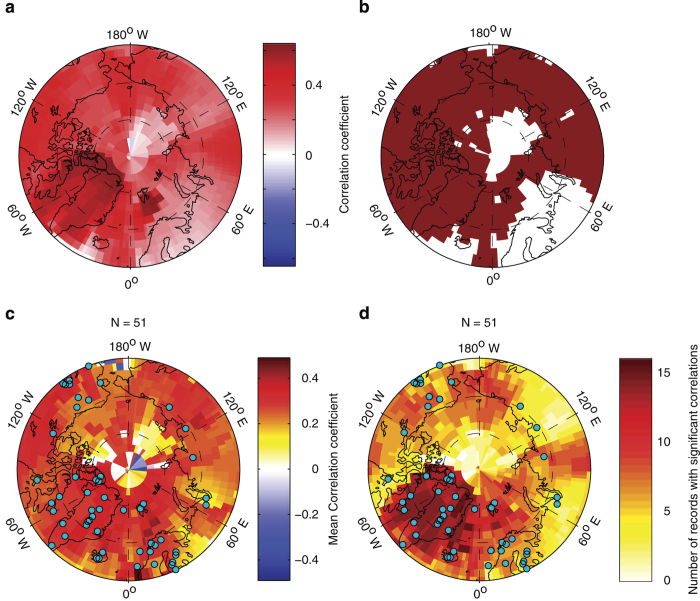
Spatiotemporal relation between annual, instrumental temperature and the PAGES Arctic 2k database. (**a**) Correlation coefficient between observed temperature at each grid cell and the revised Arctic 2k temperature reconstruction between AD 1880 and 2000. (**b**) Grid cells with significant (*P*<0.05; corrected for serial autocorrelation) correlations in (**a**) are shown in dark red. (**c**) Mean significant correlation coefficient at each grid cell for all records in the database AD 1880 and 2000. (**d**) Number of records with significant correlations (as in **b**) at each grid cell, this is equivalent to the number of records used at each grid cell to calculate the mean correlation coefficients in C. Location of records in database shown as light blue dots in C and D.

This analysis shows that the revised PAGES Arctic 2k temperature reconstruction does an excellent job of capturing observed temperature variability in the Arctic, with significant (*P*<0.05) correlations over most the Arctic (Figure 3a,b). This is consistent with patterns observed from the summary of individual record field correlations (Figure 3c,d), although several of the sites demonstrate insignificant correlations over much or even all of the Arctic (Supplementary Figure S1). These records are typically those with low resolution and time uncertainty, which confounds this analyis. The interpretation of temperature sensitivity at these sites is derived from expert understanding of the system, rather than statistical comparison with instrumental data. In both the PAGES Arctic 2k temperature reconstruction, and as a whole from the individual sites, the highest correlations were calculated over northeast Canada and Greenland, where data density is highest. Interestingly, despite strong data coverage, and several sites with strong local correlations (Supplementary Figure S1), the temperature variability in Fennoscandia is not particularly well represented in the database. This may be due to out-of-phase decadal-scale temperature variability between Fennoscandia and the western part of the North Atlantic. Indeed, instrumental temperatures from near Greenland and northeastern Canada are poorly correlated with temperatures from Fennoscandia (Supplementary Figure S2). Some of this feature is due to the choice to compare the reconstruction to annual temperatures, thereby integrating some of the strong out-of-phase relationship that characterizes the region during the winter. However, a weaker, but similar pattern is present when analyzing summer (JJA) data only (Supplementary Figure S3). We also examine how the reconstruction correlates with instrumental summer (JJA) temperatures (Supplementary Figures S4). As expected, the reconstruction correlates better with summer than annual temperatures over Fennoscandia, however, the results are mixed elsewhere. Correlations with winter half-year (ONDJFM) temperatures strongly resemble annual correlations, but with fewer significant correlations across the Arctic. This resemblance is likely due to the dominance of winter temperature variability in the Arctic^69^. Overall, the reconstruction and records as a whole appear more representative of annual than either winter or summer temperatures. This is not because the records are sensing annual temperatures, rather, it is likely an artifact of including both summer and winter sensitive records in the compilation. Indeed, the spatial heterogeneity of the response highlights the biases introduced due to the variable seasonal response of proxy types and individual sites, and the shortcomings of index reconstructions, and highlights the need for a more nuanced consideration of spatial and seasonal variability in paleoclimate syntheses.

Finally, it should be noted that whereas these analyses are useful for quantifying some aspects of temperature sensitivity, they are poorly suited to determine the extent to which the records reflect long-term (centuries to millennia) changes in past temperature, or the stability of the modern relation back through time.

## Additional information

**How to cite this article:** McKay, N. P. and Kaufman, D. S. An extended Arctic proxy temperature database for the past 2,000 years. *Sci. Data* 1:140026 doi: 10.1038/sdata.2014.26 (2014).

## Supplementary Material



Supplementary Information

## Figures and Tables

**Table 1:** Summary of sites and proxy records in the PAGES Arctic 2k v1.1 database.

**Pages ID**	**Country/Region**	**Site**	**Lat (°N)**	**Long (°E)**	**Archive type**	**Proxy measurement**	**Oldest (AD)**	**Youngest (AD)**	**Resolution (year)**	**Seasonality**	**Ref**
Arc_1	Alaska	Blue lake	68.1	−150.5	Lake sediment	Varve thickness	730	2005	1	Summer	^23^
Arc_2	Central Russia	Avam-Taimyr	72.0	101.0	Tree ring	Ring width	−100	2003	1	Jun–Jul	^30^
Arc_3	Central Russia	Yamal	67.5	70.0	Tree ring	Ring width	1	1996	1	May–Jul	^30^
Arc_4	Canada	Lower Lake Murray	81.4	−69.5	Lake sediment	Mass accumulation rate	−3236	2004	1	Melt Season	^31^
Arc_5	Greenland	Camp Century	77.2	−61.1	Ice core	d18O	1242	1967	1	Annual	^32^
Arc_6	Alaska	Seward Peninsula	65.2	−162.2	Tree ring	Ring width	1288	2002	1	Mean summer	^33^
Arc_7	Alaska	Gulf of Alaska	61.0	−146.6	Tree ring	Ring width	800	2010	1	Feb–Aug	^19^
Arc_8	Canada	Yukon	67.9	−140.7	Tree ring	Ring width	1177	2002	1	Annual	^34^
Arc_9	Canada	Coppermine River	67.1	−155.6	Tree ring	Ring width	1048	2003	1	Jun–jul	^24^
Arc_10	Central Russia	Polar Urals	66.8	65.8	Tree ring	Maximum density	778	1990	1	Jun–jul	^35^
Arc_11	Greenland	GISP2	72.1	−38.1	Ice core	d18O	818	1987	1	Annual	^36^
Arc_12	Scandinavia	Torneträsk	68.3	19.6	Tree ring	Ring Width	−39	2010	1	Apr–Aug	^18^
Arc_13	Scandinavia	Jämtland	63.5	15.5	Tree ring	Maximum density	1107	2007	1	Apr–Sep	^37^
Arc_14	Scandinavia	Lake Lehmilampi	63.6	29.1	Lake sediment	Varve thickness	1	1800	1	Winter	^38^
Arc_15	Scandinavia	Lapland	69.0	25.0	Tree ring	Ring width	0	2005	1	Summer	^39^
Arc_16	Eastern Russia	Indigurka	69.5	147.0	Tree ring	Ring width, STD	1259	1994	1	Early summer	^40^
Arc_17	North Atlantic	Lomonosovfonna	78.9	17.4	Ice core	d18O	1400	1997	1	Dec–Feb	^17^
Arc_18	North Atlantic	Austfonna	79.8	24.0	Ice core	d18O	1400	1998	1	Dec–Feb	^41^
Arc_19	Scandinavia	Forfjorddalen 2	69.1	17.2	Tree ring	Ring width	877	1994	1	Jul–Aug	^25^
Arc_20	Arctic Canada	Lake C2	82.1	−77.2	Lake sediment	Varve thickness	−1309	1987	1	Summer	^42^
Arc_22	North Atlantic	Hvítárvatn	64.6	−19.8	Lake sediment	Varce thickness	−981	2002	1	Summer	^28^
Arc_23	Alaska	Iceberg Lake	60.8	−143.0	Lake sediment	Varve thickness	442	1998	1	Jun–Aug	^43^
Arc_24	Eastern Russia	Lower Lena River	70.7	125.9	Tree ring	Ring width, ARS	1408	1994	1	June	^26^
Arc_25	Arctic Canada	Donard Lake	66.7	−61.4	Lake sediment	Thickness	752	1992	1	Summer	^44^
Arc_26	Scandinavia	Lake Nautajärvi	61.8	24.7	Lake sediment	Organic matter	0	1800	1	Summer	^45^
Arc_27	Greenland	B16	73.9	−37.6	Ice core	d18O	1478	1992	1	Annual	^46^
Arc_28	Greenland	B18	76.6	−36.4	Ice core	d18O	871	1992	1	Annual	^46^
Arc_29	Greenland	B21	80.0	−41.1	Ice core	d18O	1397	1993	1	Annual	^46^
Arc_30	Arctic Canada	Big Round Lake	69.9	−68.8	Lake sediment	Varve thickness	971	2003	1	Jul–Sep	^47^
Arc_31	Scandinavia	Lake Korttajärvi	62.3	25.7	Lake sediment	X-ray density	0	1720	1	Spring-summer	^12^
Arc_32	Greenland	NGRIP1	75.1	−42.3	Ice core	d18O	0	1995	1	Annual	^48^
Arc_33	Arctic Canada	Agassiz Ice Cap	80.7	−73.1	Ice core	d18O	0	1972	1	Annual	^49^
Arc_34	Greenland	Crête	71.1	−37.3	Ice core	d18O	553	1973	1	Annual	^50^
Arc_35	Greenland	Dye-3	65.2	−43.8	Ice core	d18O	1	1979	1	Annual	^50^
Arc_36	Greenland	GRIP	72.6	−37.6	Ice core	d18O	1	1979	1	Annual	^50^
Arc_37	North Atlantic	Iceland	64.8	−18.4	Historic	Ice cover	945	1935	30	Winter	^51^
Arc_38	North Atlantic	MD95-2011	67.0	7.6	Marine sediment	Diatoms	−4076	1995	10	Aug	^52^
Arc_39	North Atlantic	MD95-2011	67.0	7.6	Marine sediment	Alkenone	−6540	1440	28	Summer	^53^
Arc_40	Alaska	Moose lake	61.3	−143.6	Lake sediment	Midge assemblages	−4058	1970	38	Jul	^54^
Arc_41	Alaska	Hudson Lake	61.9	−145.7	Lake sediment	Midge assemblages	−7640	1976	50	Jul	^55^
Arc_42	Alaska	Screaming Lynx Lake	66.1	−145.4	Lake sediment	Midge assemblages	−8661	1993	36	Jul	^55^
Arc_43	Greenland	Lake Braya So	67.0	−50.7	Lake sediment	Uk37	−4169	2005	29	Summer	^56^
Arc_44	Arctic Canada	Devon Ice Cap	75.3	−82.5	Ice core	proxy	−727	1973	5	Annual	^57^
Arc_45	Arctic Canada	Penny Ice Cap	67.3	−66.8	Ice core	d18O	−9733	1992	25	Annual	^57^
Arc_47	North Atlantic	MD99-2275	66.6	−17.4	Marine sediment	Diatoms	−36	1949	20	Summer	^58^
Arc_48	Alaska	Lone Spruce Pond	60.0	−159.1	Lake sediment	BSi	###	2005	10	Growing Season	^29^
Arc_49	Scandinavia	Okshola cave	67.0	15.0	Speleothem	d18O	−5565	1997	32	Annual	^59^
Arc_50	Scandinavia	Lake Hampträsk	60.3	25.4	Lake sediment	Chironomids	1330	2000	15	July	^60^
Arc_51	Scandinavia	Lake Pieni-Kauro	64.3	30.1	Lake sediment	Chironomids	470	1990	43	July	^61^
Arc_52	North Atlantic	Lake Igaliku	61.0	−45.4	Lake sediment	Pollen accumulation	−7577	2001	58	Summer	^27^
Arc_53	Arctic Canada	Penny Ice Cap	67.3	−66.8	Ice core	Ice melt	−966	1984	25	Summer	^62^
Arc_54	Canada	Lake 4	65.1	−83.8	Lake sediment	Chironomid	814	1997	44	August	^63^
Arc_55	North Atlantic	P1003	63.8	5.3	Marine sediment	d18O	−5931	1998	8	Annual	^64^
Arc_57	North Atlantic	MD99-2275	66.6	−17.4	Marine sediment	Alkenone	−2549	2001	4	July	^65^
Arc_58	North Atlantic	MSM5/5-712	78.9	6.8	Marine sediment	Planktic foraminifers	−94	2007	41	Jul–Sep	^66^
Arc_59	Greenland	Renland	71.3	−26.7	Ice core	d18O	0	1980	5	Annual	^49^
Note: Updates to PAGES 2k Consortium^1^ Arctic temperature reconstruction. Arc_1: Restricted to temperature sensitive section after 730 AD. Arc_7: Updated to Wiles *et al.* ^19^ Arc_9: Corrected coordinates and restricted to temperature-sensitive section. Arc_12: Updated to Melvin *et al.* ^18^ Arc_17: Updated to Divine *et al.* ^17^ Arc_19: Restricted to temperature sensitive section. Arc_21: Omitted (not temperature sensitive). Arc_22: Corrected temperature relation. Arc_24: Restricted to temperature sensitive section. Arc_46: Omitted (not temperature sensitive). Arc_48: Corrected 50-year age offset. Arc_52: removed two most recent values due to anthropogenic fertilizer influence. Arc_56: Omitted (not temperature sensitive).											
