# Supplementary material for: An extended Arctic proxy temperature database for the past 2,000 years
Source: Sci Data. 2014 Aug 19;1:140026. doi: 10.1038/sdata.2014.26 (PMC4322576; doi:10.1038/sdata.2014.26)
Supplement: Supplementary Information [file sdata201426-s2.pdf]

Supplementary Materials

Figure S1. Correlation and significance maps for the 39 sites in the dataset with at least 8 data points overlapping the instrumental record (1880-2000). For all sites, the map on the left panel illustrates the correlation coefficient between observed temperature at each grid cell and the corresponding temperature-sensitive proxy measurements at that site. For the sites with author-interpreted negative relationships with temperature (i.e., lower proxy values correspond to warmer temperatures), the records are inverted prior to correlation. Grid cells with significant ($p<0.05$; corrected for serial autocorrelation) correlations in are shown in dark red on the right panel. The location of the sites are marked with a black star.

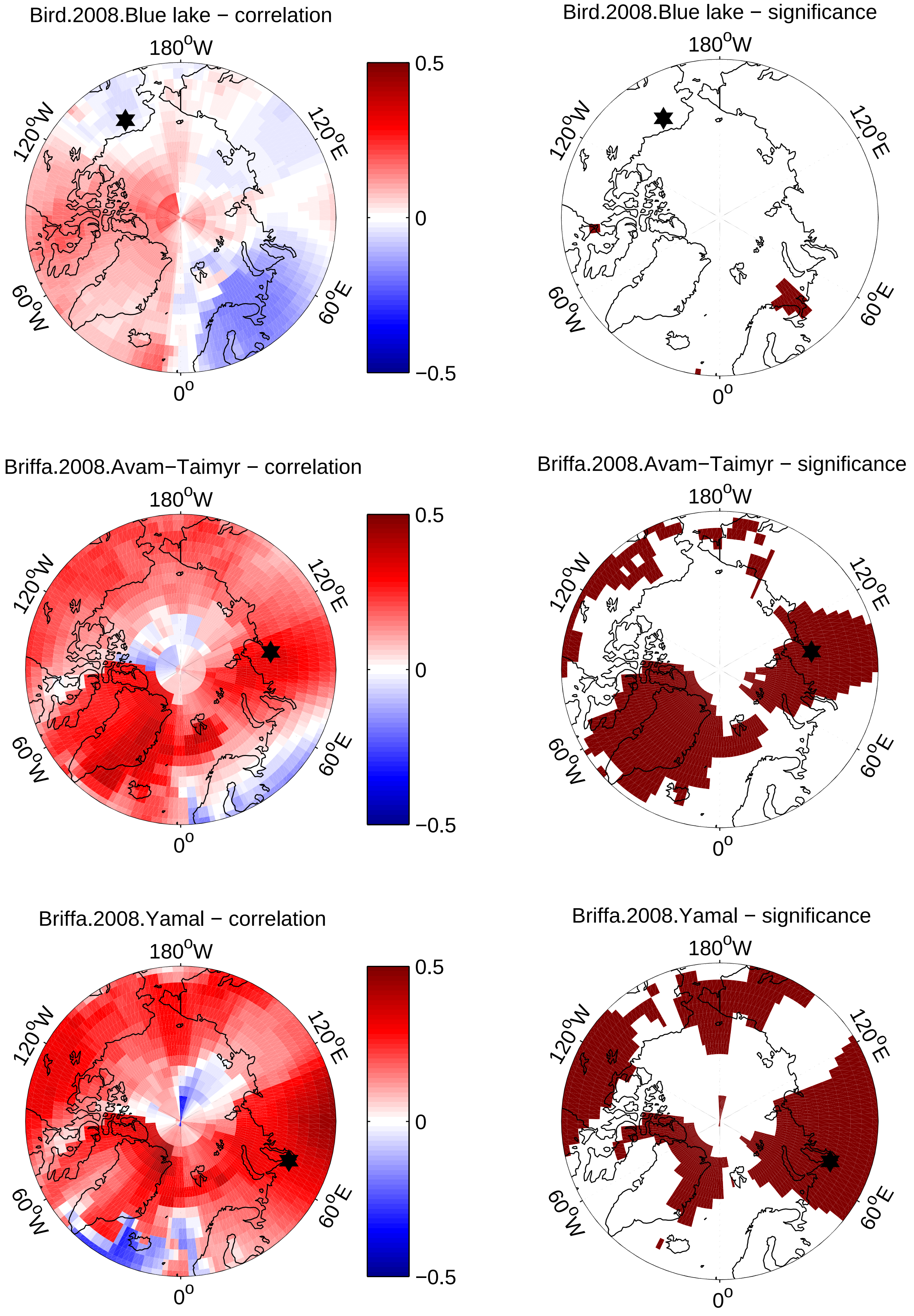
Bird.2008.Blue lake − correlation
Bird.2008.Blue lake − significance
Briffa.2008.Avam−Taimyr − correlation
Briffa.2008.Avam−Taimyr − significance
Briffa.2008.Yamal − correlation
Briffa.2008.Yamal − significance
180°W
120°W
120°E
60°W
60°E
0°
0.5
0
−0.5

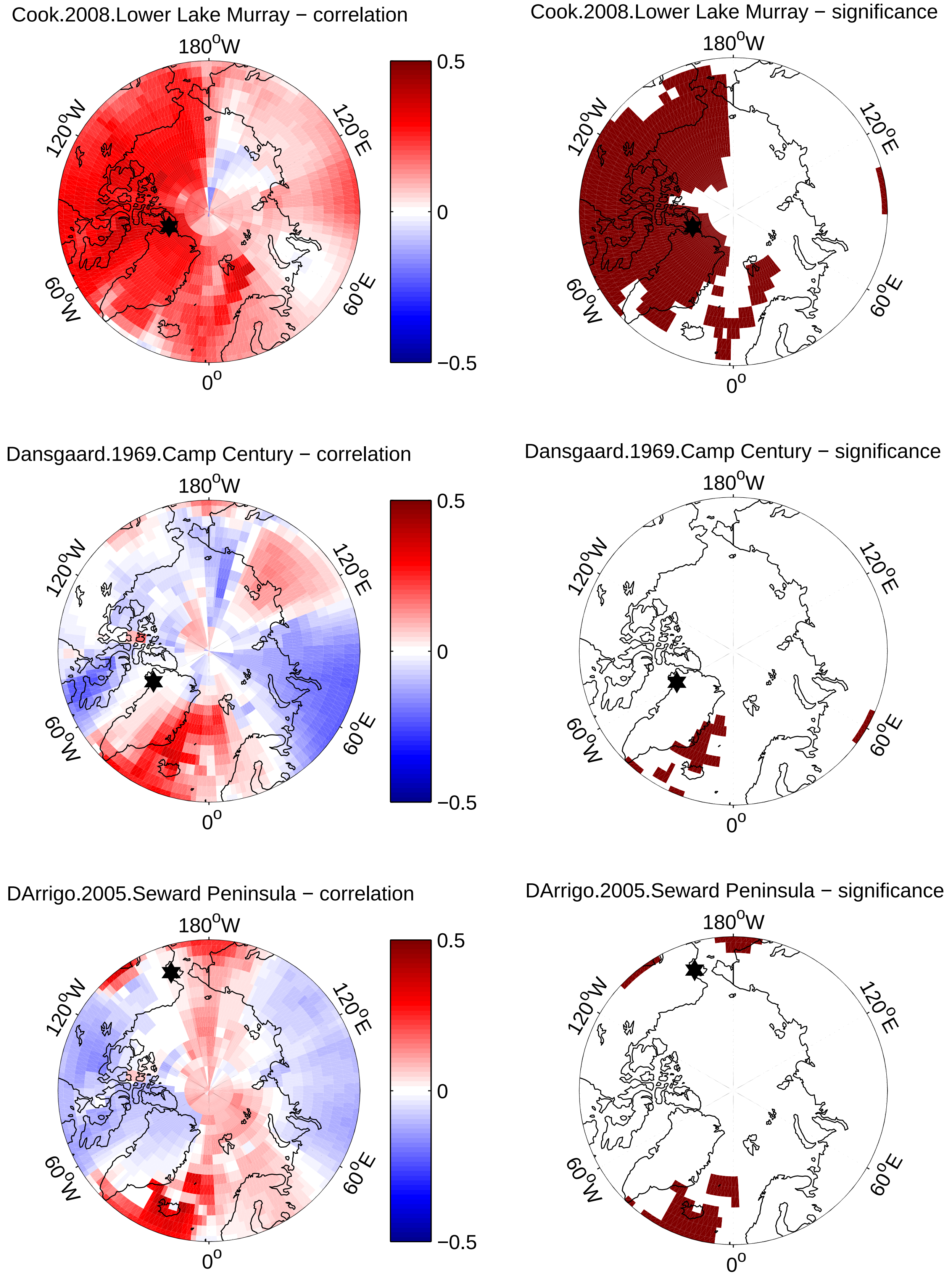
Cook.2008.Lower Lake Murray − correlation
Cook.2008.Lower Lake Murray − significance
Dansgaard.1969.Camp Century − correlation
Dansgaard.1969.Camp Century − significance
DArrigo.2005.Seward Peninsula − correlation
DArrigo.2005.Seward Peninsula − significance
180$^o$W
120$^o$W
120$^o$E
60$^o$W
60$^o$E
0$^o$
0.5
0
−0.5

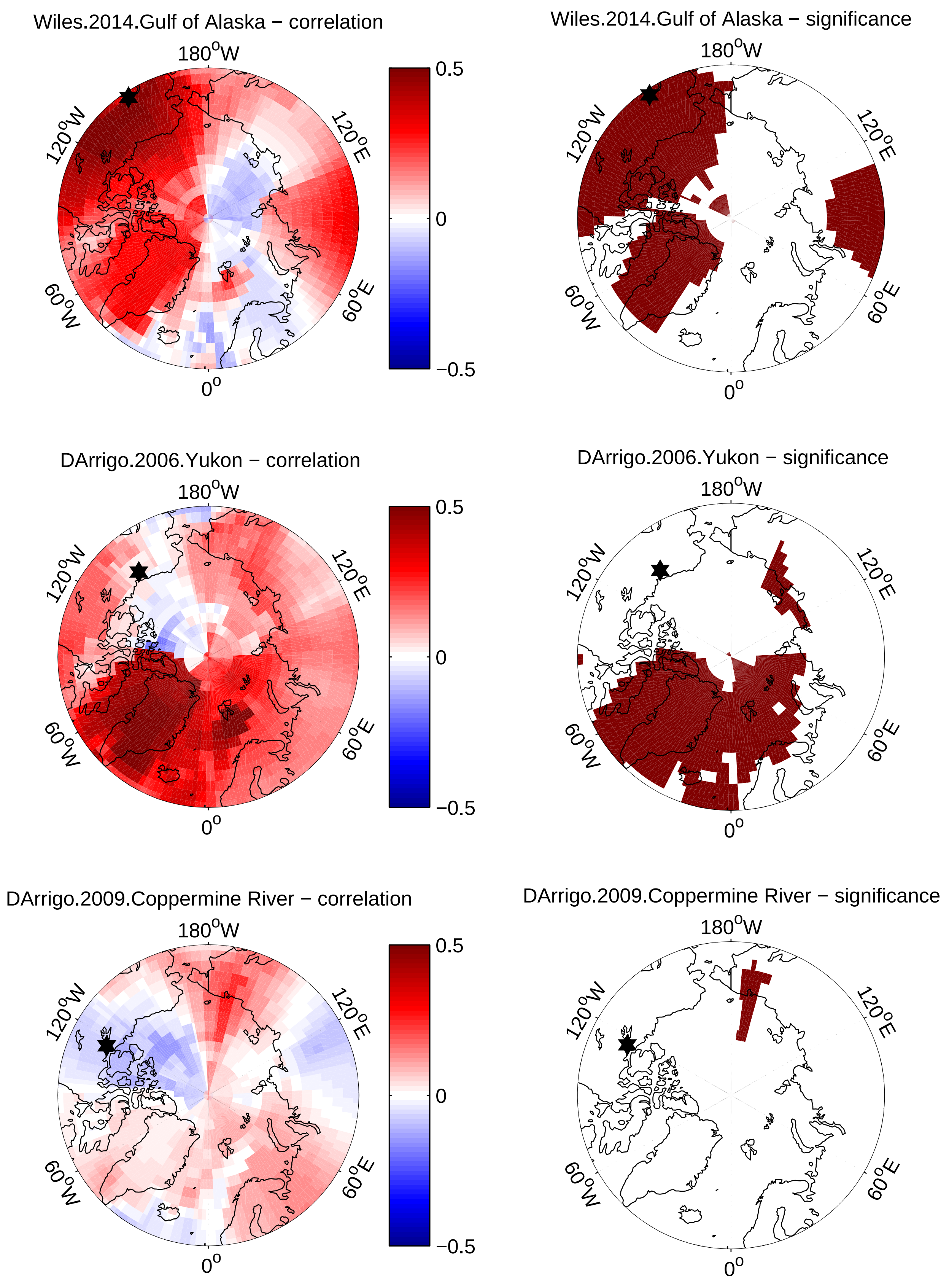

Wiles.2014.Gulf of Alaska − correlation
Wiles.2014.Gulf of Alaska − significance
DArrigo.2006.Yukon − correlation
DArrigo.2006.Yukon − significance
DArrigo.2009.Coppermine River − correlation
DArrigo.2009.Coppermine River − significance
180°W
120°W
120°E
60°W
60°E
0°
0.5
0
−0.5

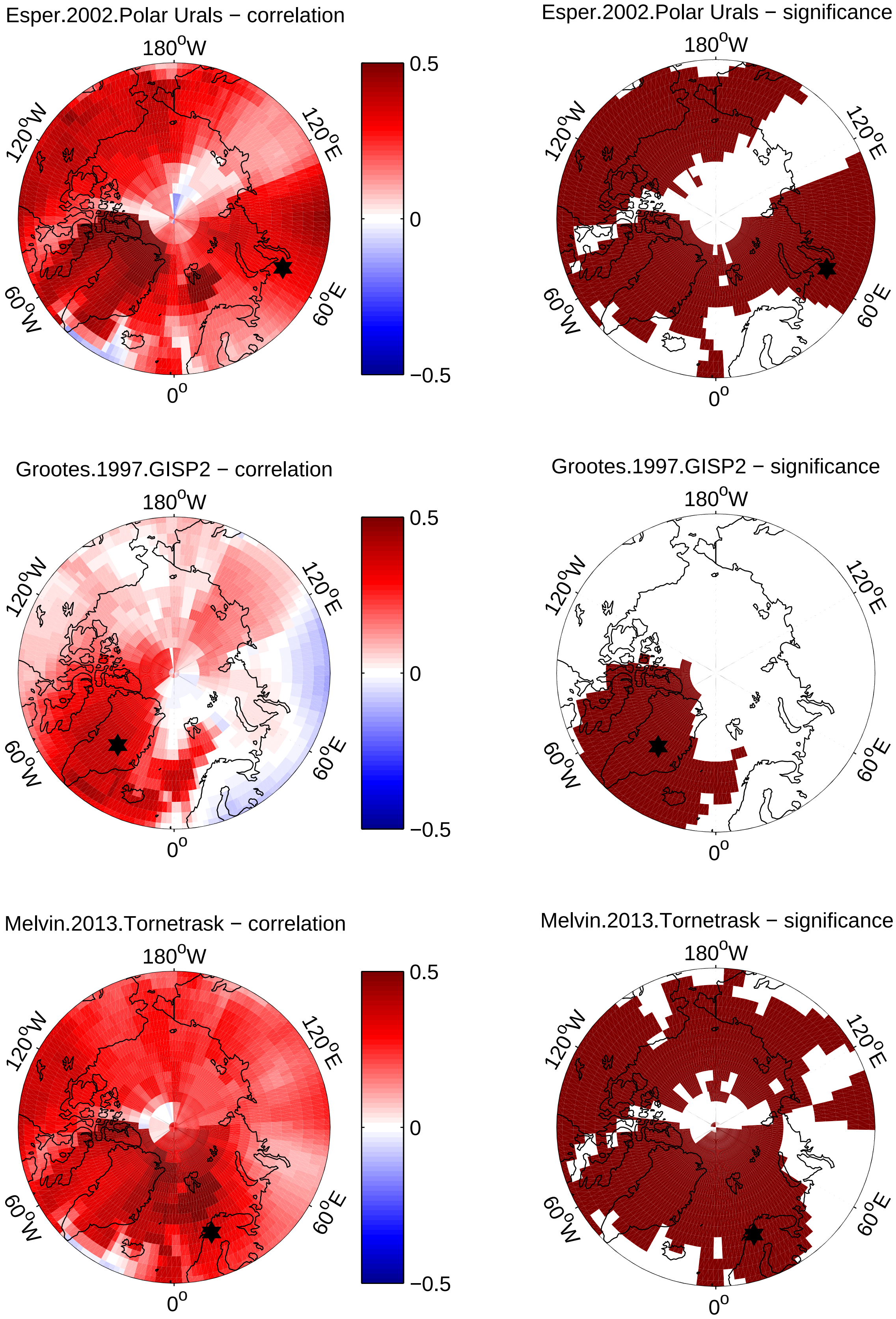
Esper.2002.Polar Urals − correlation
Esper.2002.Polar Urals − significance
Grootes.1997.GISP2 − correlation
Grootes.1997.GISP2 − significance
Melvin.2013.Tornetrask − correlation
Melvin.2013.Tornetrask − significance
180°W
120°W
120°E
60°W
60°E
0°
0.5
0
−0.5

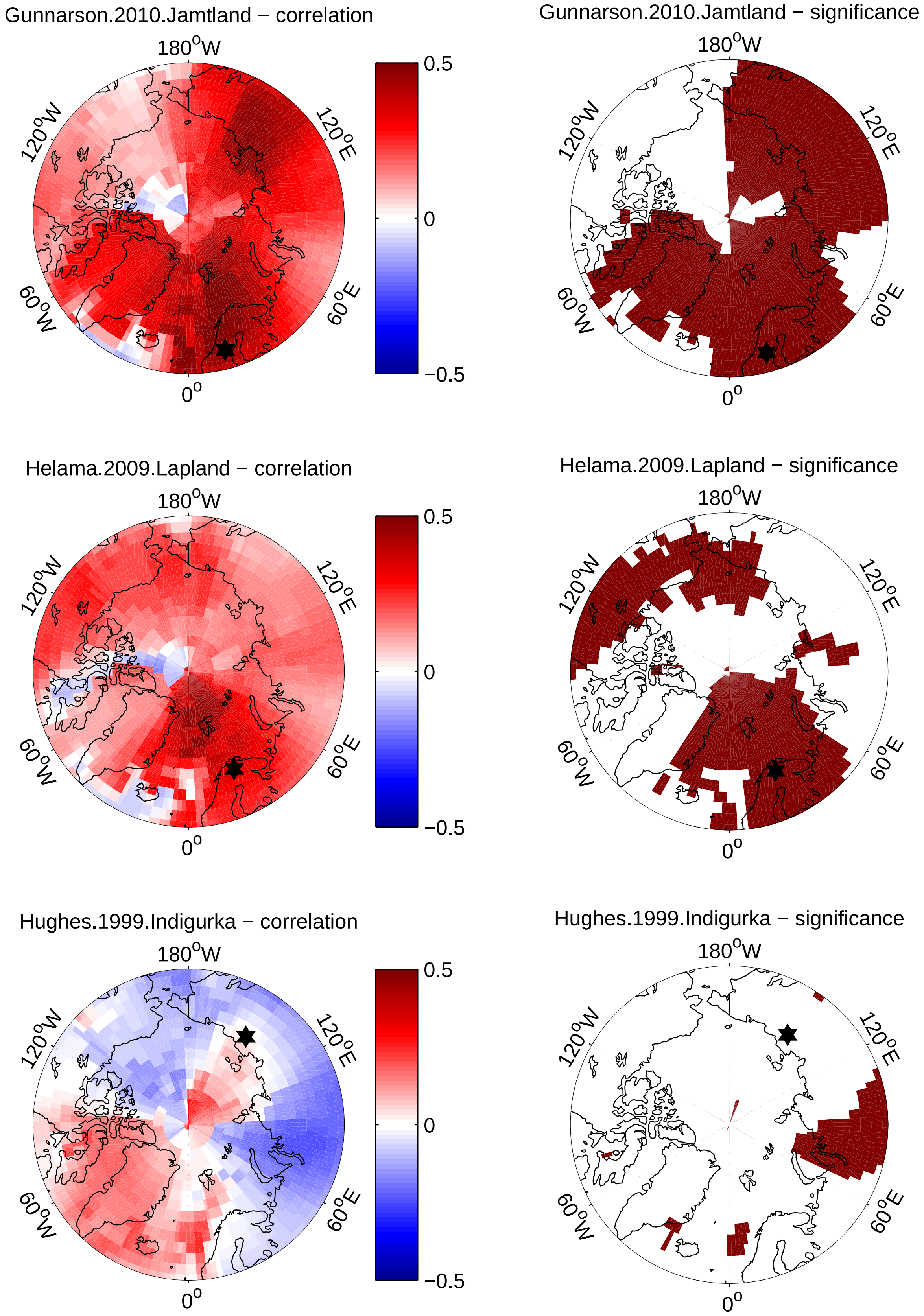
Gunnarson.2010.Jamtland − correlation
Gunnarson.2010.Jamtland − significance
Helama.2009.Lapland − correlation
Helama.2009.Lapland − significance
Hughes.1999.Indigurka − correlation
Hughes.1999.Indigurka − significance
180$^{o}$W
120$^{o}$W
120$^{o}$E
60$^{o}$W
60$^{o}$E
0$^{o}$
0.5
0
−0.5

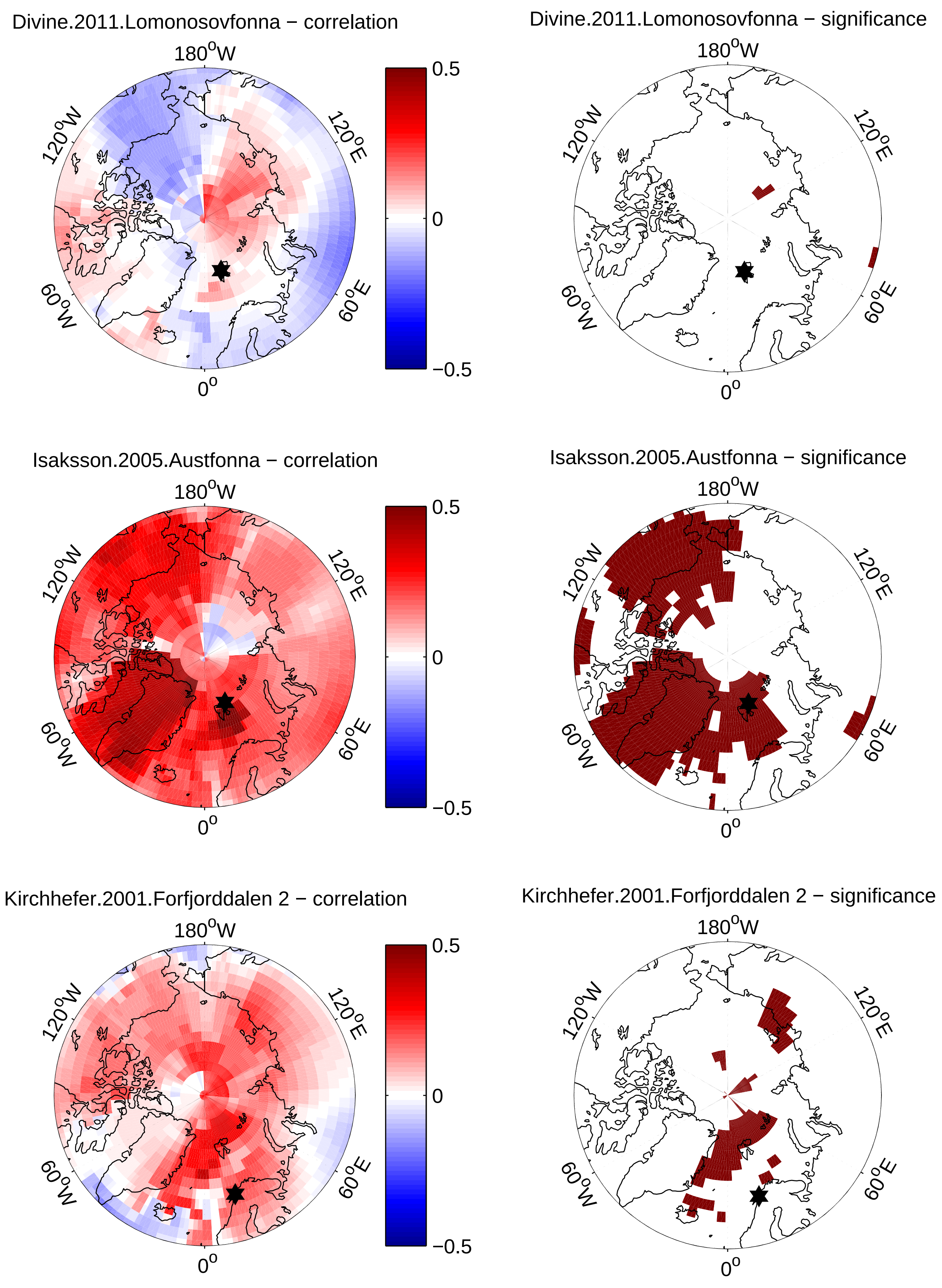
Divine.2011.Lomonosovfonna − correlation
180°W
120°W
120°E
60°W
60°E
0°
0.5
0
−0.5
Divine.2011.Lomonosovfonna − significance
180°W
120°W
120°E
60°W
60°E
0°
Isaksson.2005.Austfonna − correlation
180°W
120°W
120°E
60°W
60°E
0°
0.5
0
−0.5
Isaksson.2005.Austfonna − significance
180°W
120°W
120°E
60°W
60°E
0°
Kirchhefer.2001.Forfjorddalen 2 − correlation
180°W
120°W
120°E
60°W
60°E
0°
0.5
0
−0.5
Kirchhefer.2001.Forfjorddalen 2 − significance
180°W
120°W
120°E
60°W
60°E
0°

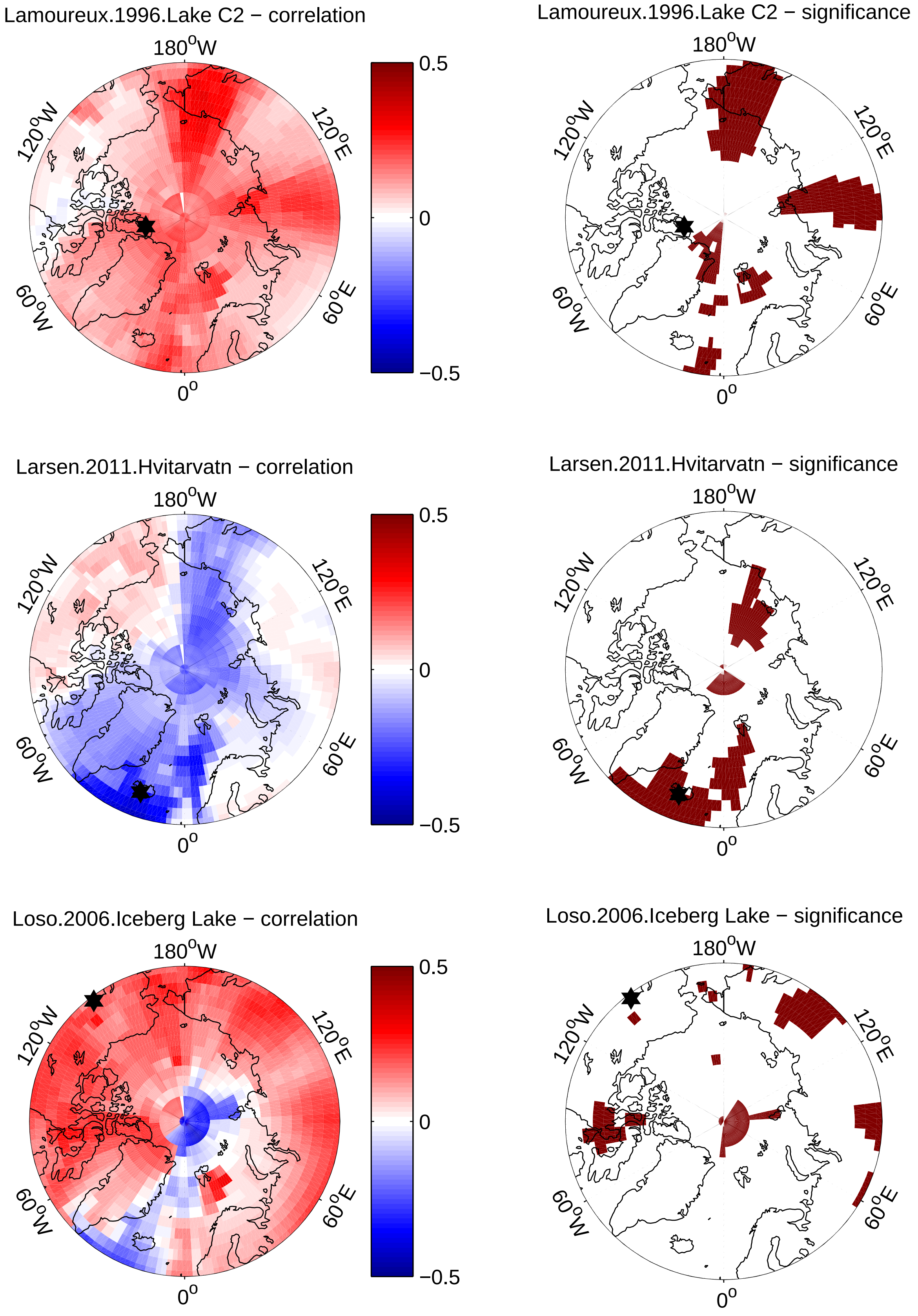
Lamoureux.1996.Lake C2 − correlation
180°W
120°W
120°E
60°W
60°E
0°
0.5
0
−0.5
Lamoureux.1996.Lake C2 − significance
180°W
120°W
120°E
60°W
60°E
0°
Larsen.2011.Hvitarvatn − correlation
180°W
120°W
120°E
60°W
60°E
0°
0.5
0
−0.5
Larsen.2011.Hvitarvatn − significance
180°W
120°W
120°E
60°W
60°E
0°
Loso.2006.Iceberg Lake − correlation
180°W
120°W
120°E
60°W
60°E
0°
0.5
0
−0.5
Loso.2006.Iceberg Lake − significance
180°W
120°W
120°E
60°W
60°E
0°

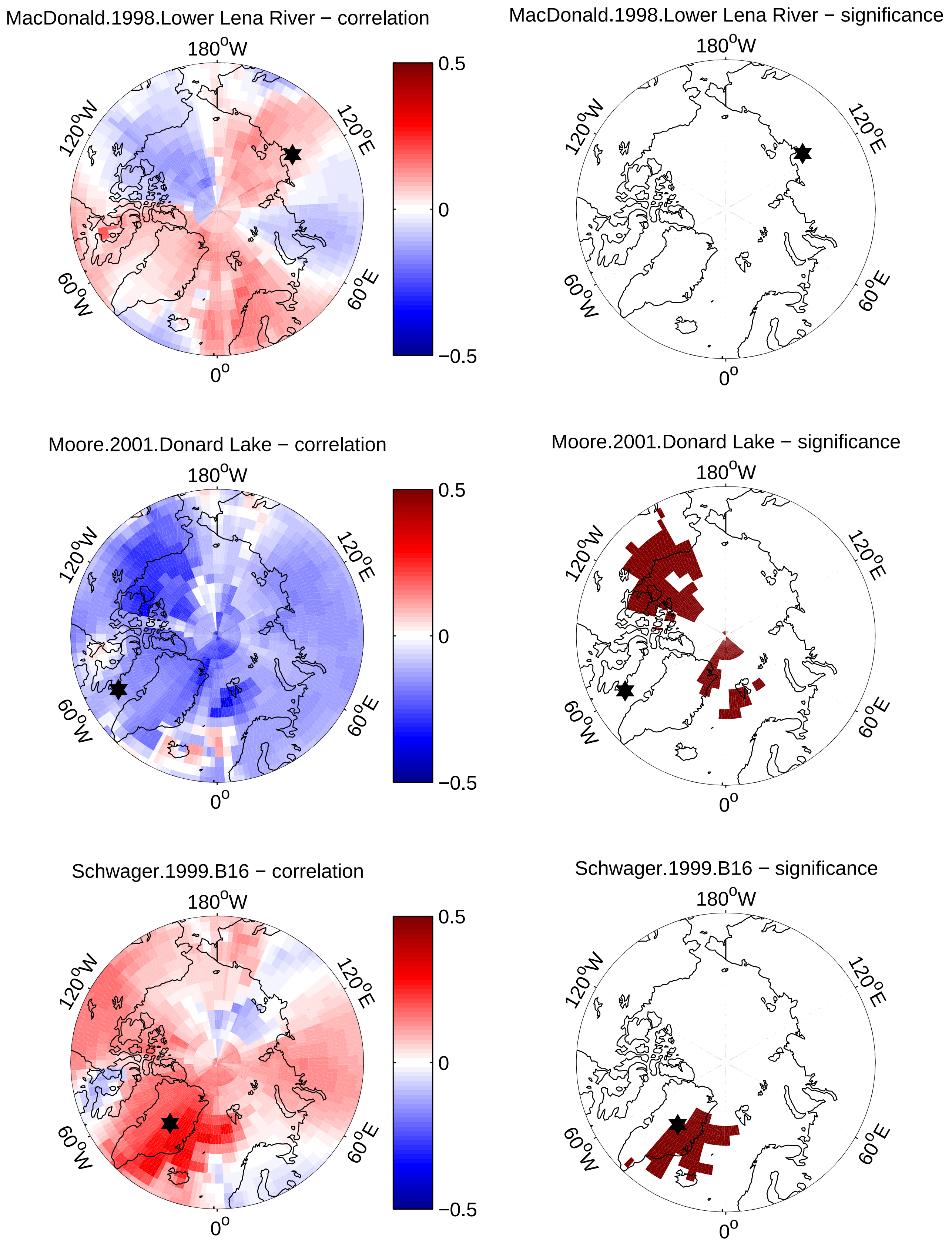
MacDonald.1998.Lower Lena River − correlation
MacDonald.1998.Lower Lena River − significance
Moore.2001.Donard Lake − correlation
Moore.2001.Donard Lake − significance
Schwager.1999.B16 − correlation
Schwager.1999.B16 − significance
180°W
120°W
120°E
60°W
60°E
0°
0.5
0
−0.5

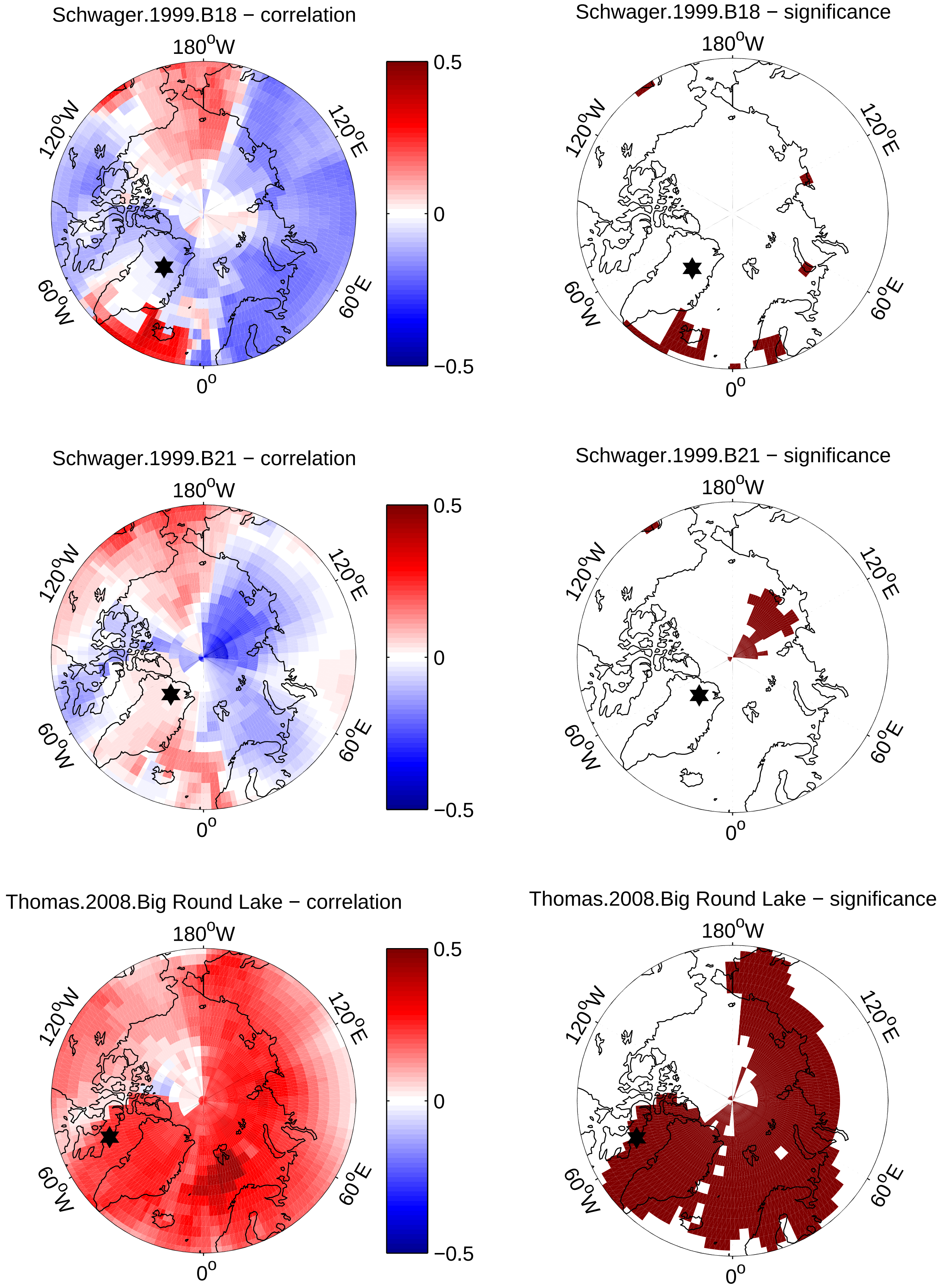
Schwager.1999.B18 − correlation
Schwager.1999.B18 − significance
Schwager.1999.B21 − correlation
Schwager.1999.B21 − significance
Thomas.2008.Big Round Lake − correlation
Thomas.2008.Big Round Lake − significance
180°W
120°W
120°E
60°W
60°E
0°
0.5
0
−0.5

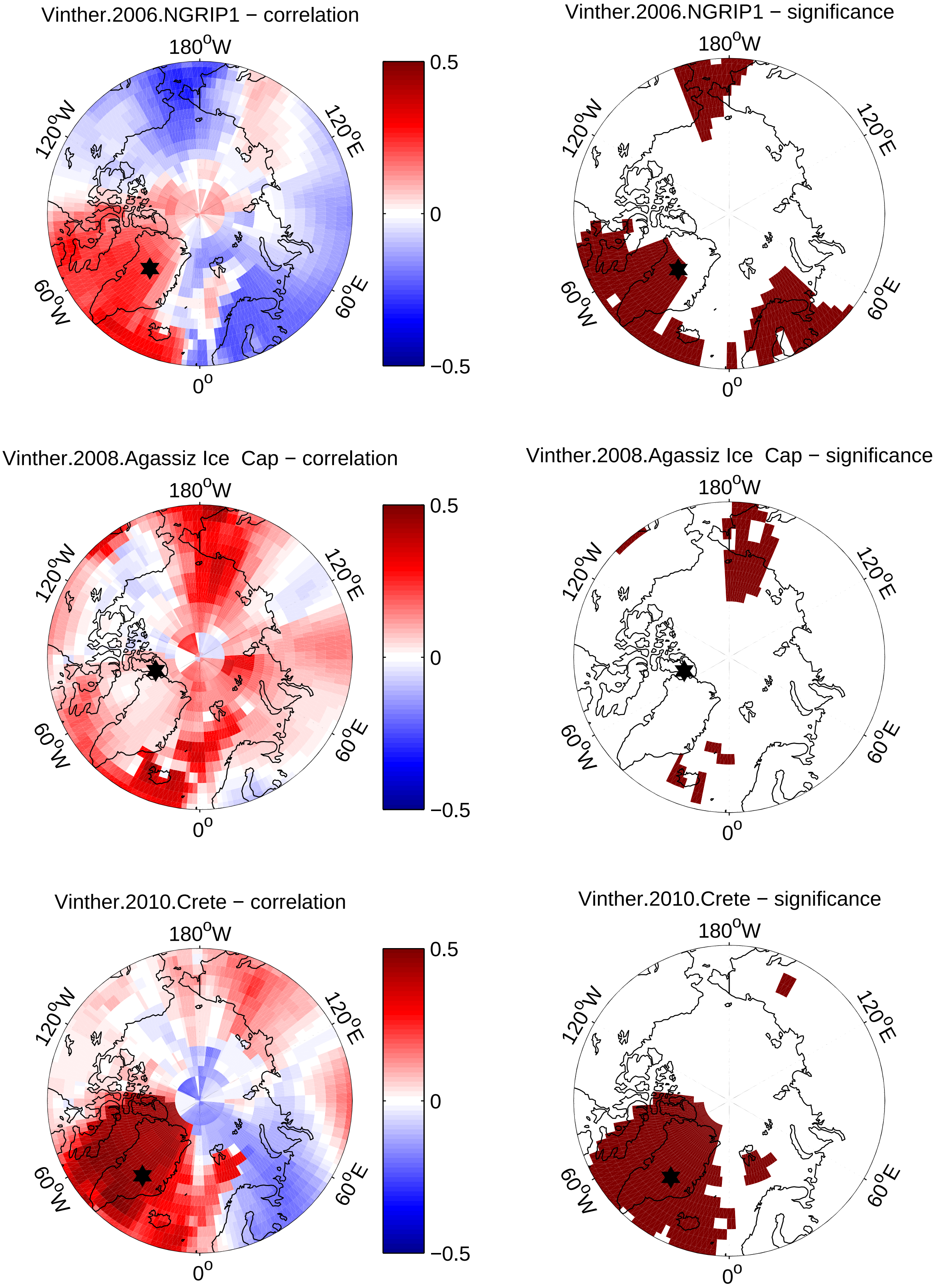
Vinther.2006.NGRIP1 − correlation
Vinther.2006.NGRIP1 − significance
Vinther.2008.Agassiz Ice Cap − correlation
Vinther.2008.Agassiz Ice Cap − significance
Vinther.2010.Crete − correlation
Vinther.2010.Crete − significance
180°W
120°W
120°E
60°W
60°E
0°
0.5
0
−0.5

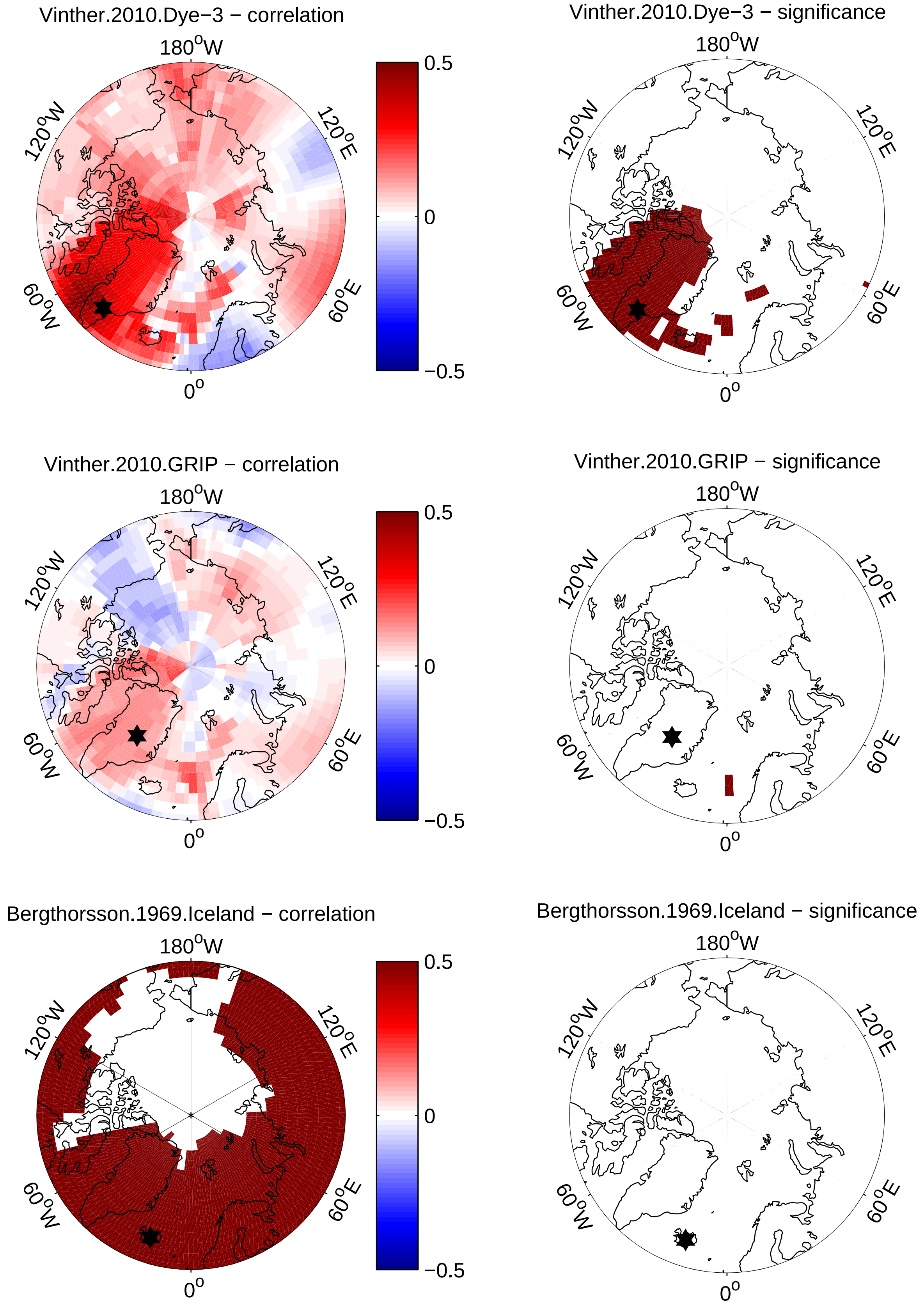

Vinther.2010.Dye−3 − correlation
Vinther.2010.Dye−3 − significance
Vinther.2010.GRIP − correlation
Vinther.2010.GRIP − significance
Bergthorsson.1969.Iceland − correlation
Bergthorsson.1969.Iceland − significance
180°W
120°W
120°E
60°W
60°E
0°
0.5
0
−0.5

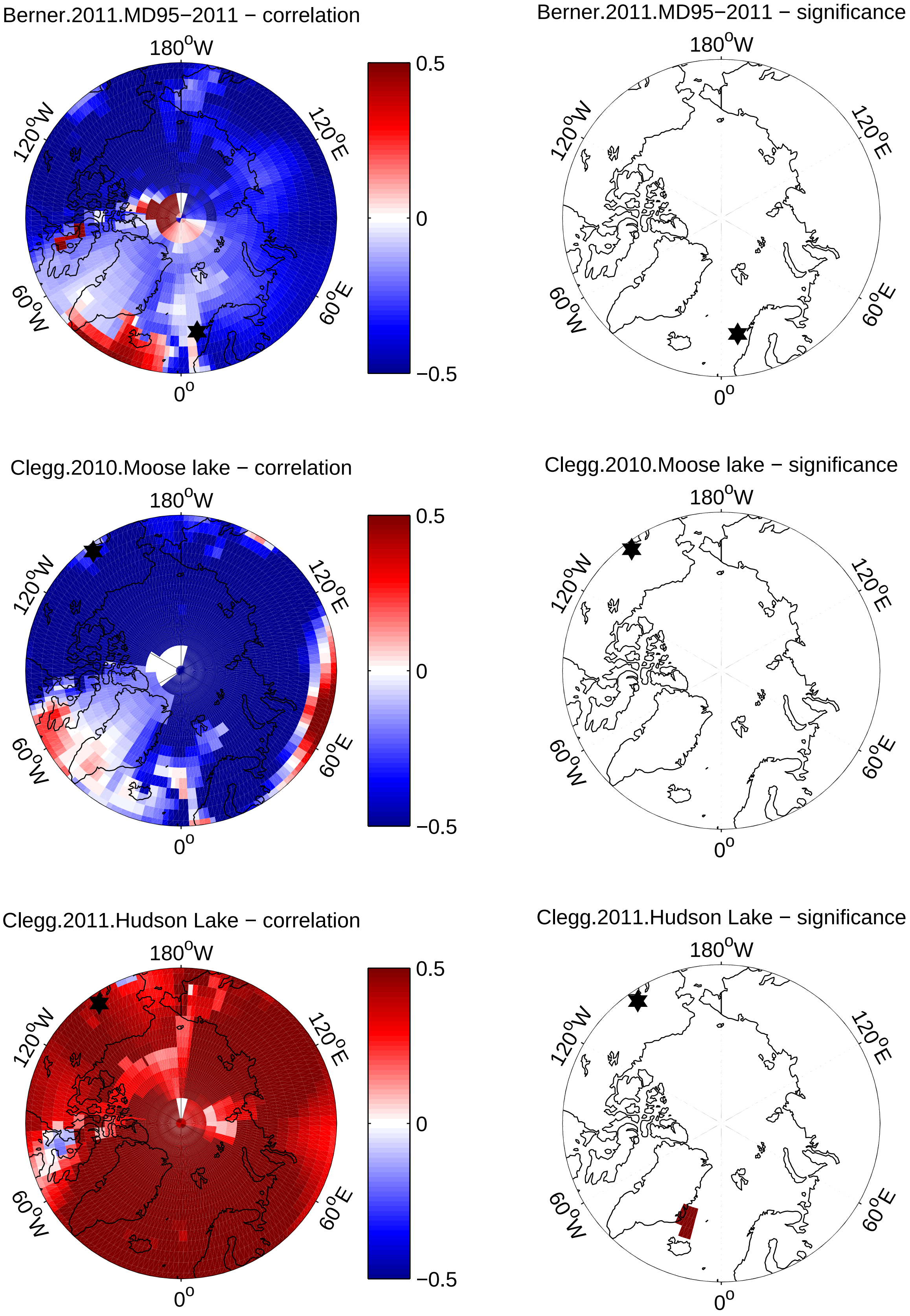

Berner.2011.MD95−2011 − correlation
Berner.2011.MD95−2011 − significance
Clegg.2010.Moose lake − correlation
Clegg.2010.Moose lake − significance
Clegg.2011.Hudson Lake − correlation
Clegg.2011.Hudson Lake − significance
180°W
120°W
120°E
60°W
60°E
0°
0.5
0
−0.5

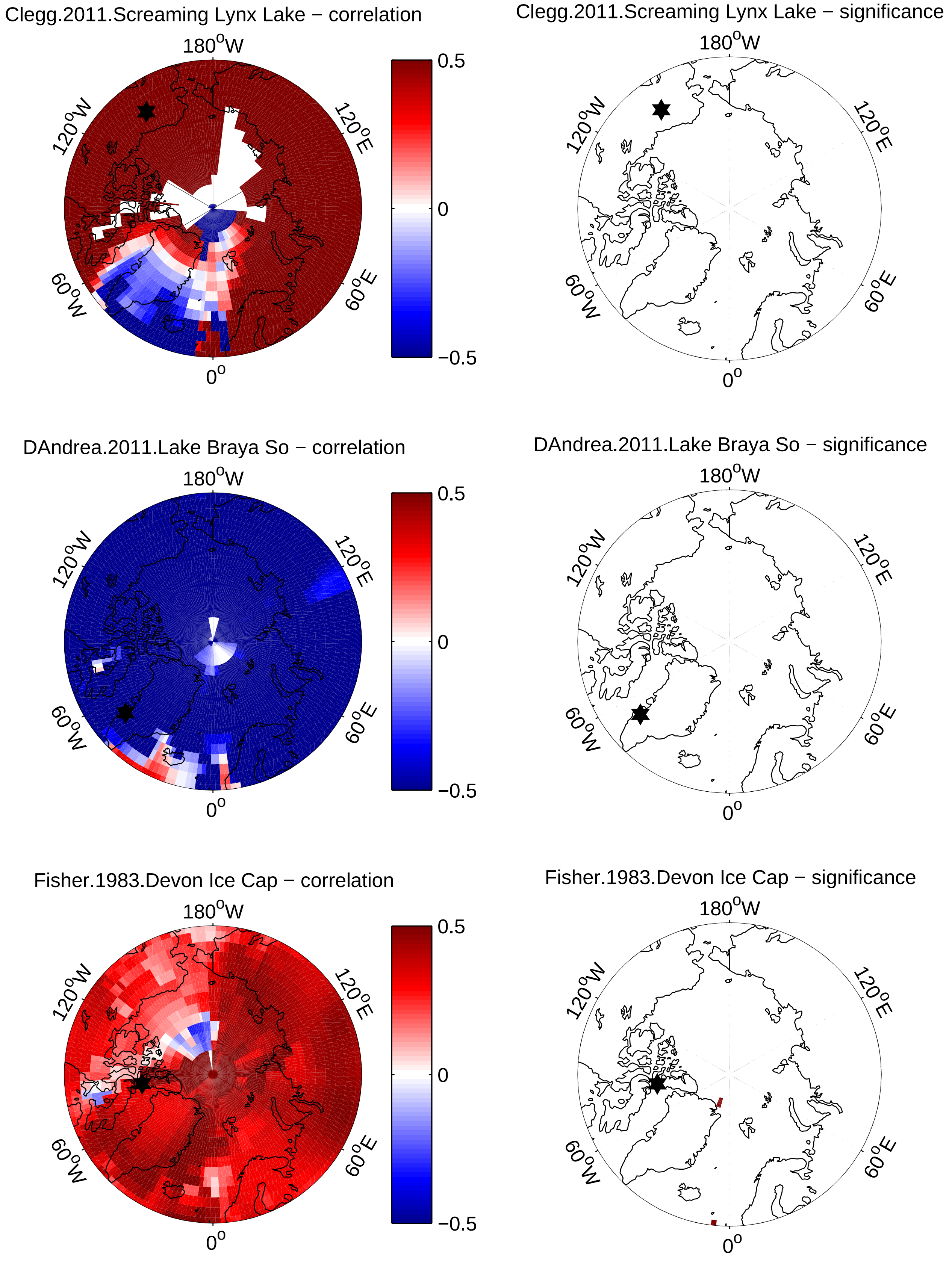
Clegg.2011.Screaming Lynx Lake − correlation
Clegg.2011.Screaming Lynx Lake − significance
DAndrea.2011.Lake Braya So − correlation
DAndrea.2011.Lake Braya So − significance
Fisher.1983.Devon Ice Cap − correlation
Fisher.1983.Devon Ice Cap − significance
180$^{o}$W
120$^{o}$W
120$^{o}$E
60$^{o}$W
60$^{o}$E
0$^{o}$
0.5
0
−0.5

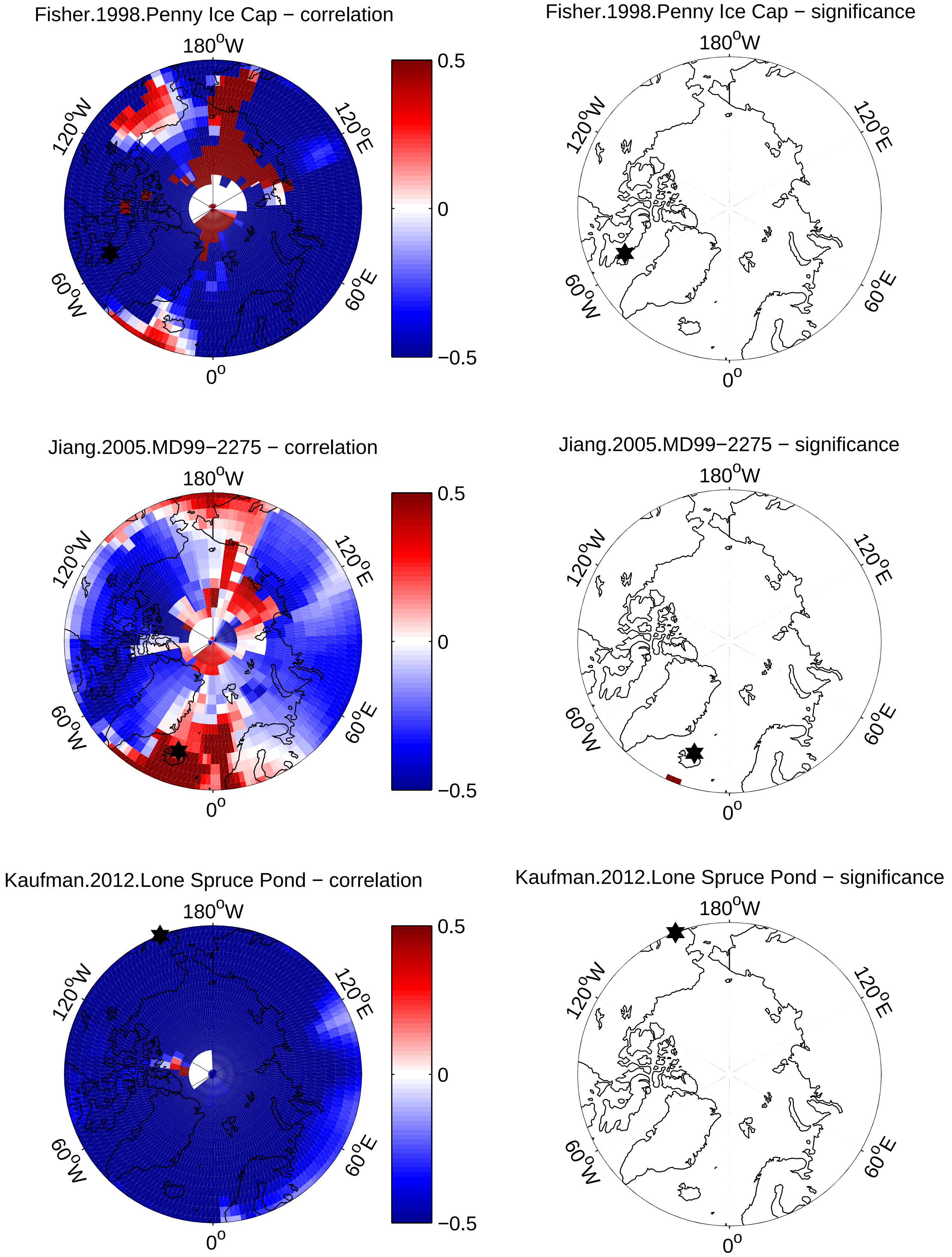

Fisher.1998.Penny Ice Cap − correlation
Fisher.1998.Penny Ice Cap − significance
Jiang.2005.MD99−2275 − correlation
Jiang.2005.MD99−2275 − significance
Kaufman.2012.Lone Spruce Pond − correlation
Kaufman.2012.Lone Spruce Pond − significance
180°W
120°W
120°E
60°W
60°E
0°
0.5
0
−0.5

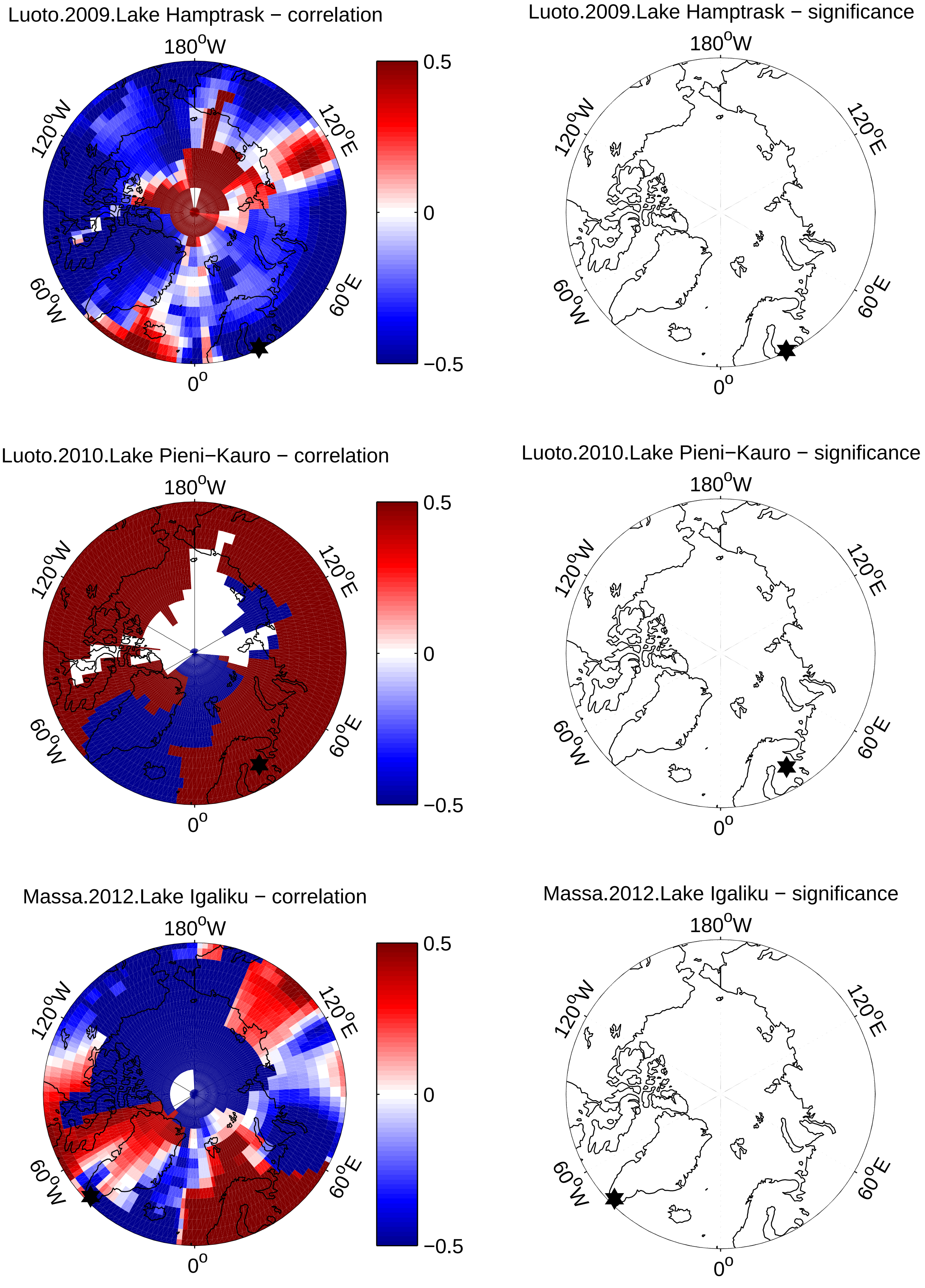
Luoto.2009.Lake Hamptrask − correlation
180°W
120°W
120°E
60°W
60°E
0°
0.5
0
−0.5
Luoto.2009.Lake Hamptrask − significance
180°W
120°W
120°E
60°W
60°E
0°
Luoto.2010.Lake Pieni−Kauro − correlation
180°W
120°W
120°E
60°W
60°E
0°
0.5
0
−0.5
Luoto.2010.Lake Pieni−Kauro − significance
180°W
120°W
120°E
60°W
60°E
0°
Massa.2012.Lake Igaliku − correlation
180°W
120°W
120°E
60°W
60°E
0°
0.5
0
−0.5
Massa.2012.Lake Igaliku − significance
180°W
120°W
120°E
60°W
60°E
0°

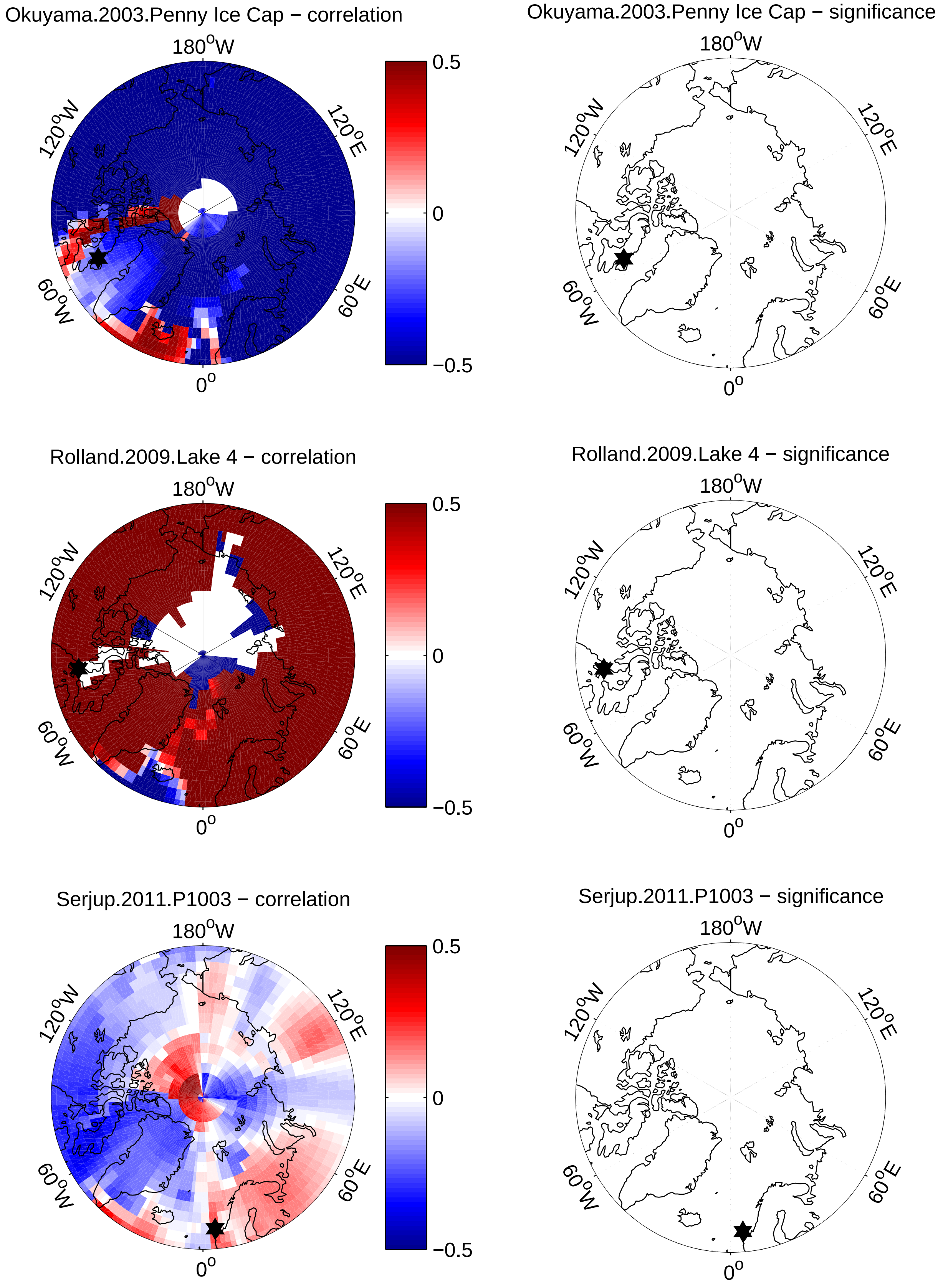

Okuyama.2003.Penny Ice Cap − correlation
Okuyama.2003.Penny Ice Cap − significance
Rolland.2009.Lake 4 − correlation
Rolland.2009.Lake 4 − significance
Serjup.2011.P1003 − correlation
Serjup.2011.P1003 − significance
180°W
120°W
120°E
60°W
60°E
0°
0.5
0
−0.5

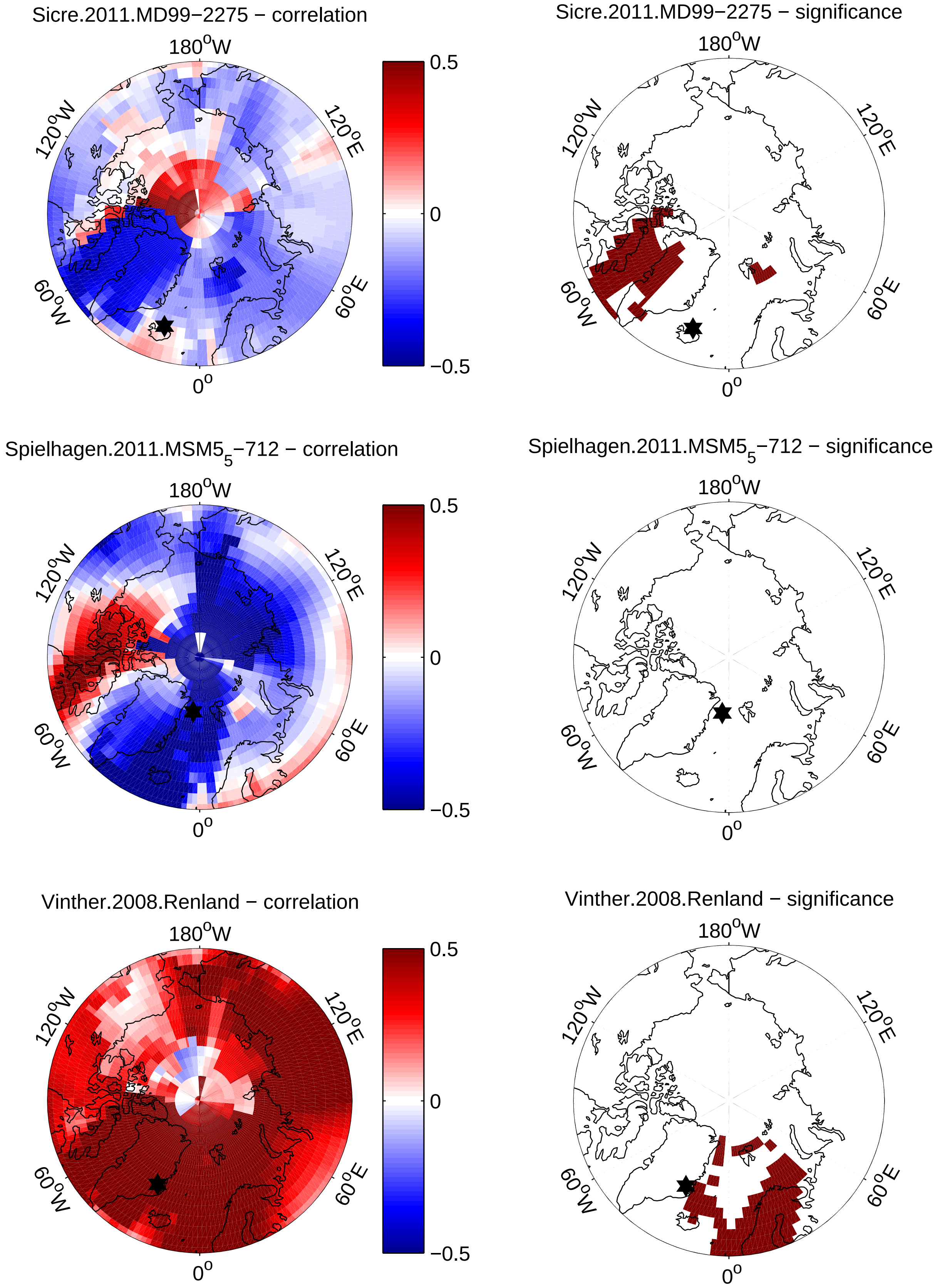

Sicre.2011.MD99−2275 − correlation
Sicre.2011.MD99−2275 − significance
Spielhagen.2011.MSM5_5−712 − correlation
Spielhagen.2011.MSM5_5−712 − significance
Vinther.2008.Renland − correlation
Vinther.2008.Renland − significance
180°W
120°W
120°E
60°W
60°E
0°
0.5
0
−0.5

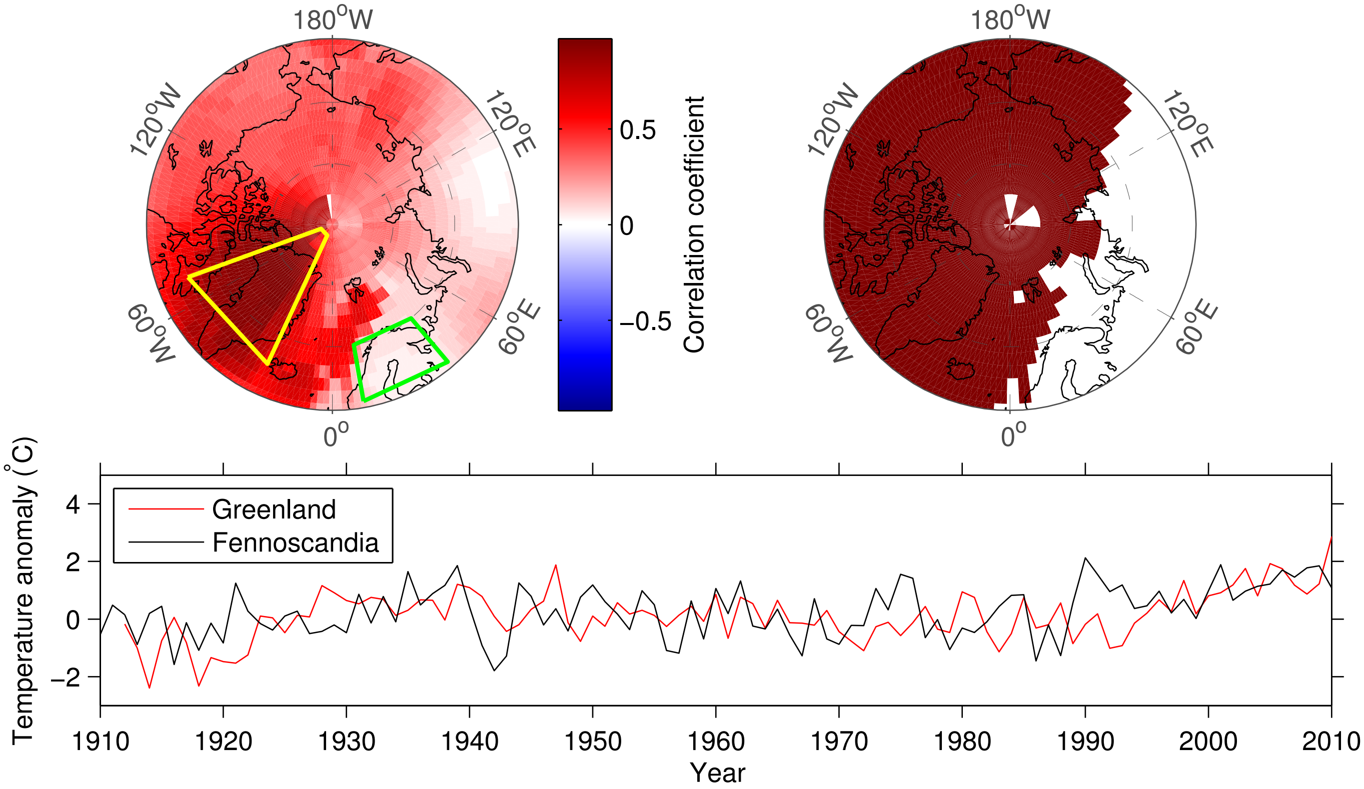


Figure S2. Upper left: correlation coefficient between area-weighted mean annual temperatures in the green box and every grid cell in the in the GISTEMP-1200 analysis (Hansen et al., 2010) from AD 1912-2010. Upper right: Spatial distribution of significant ($p < 0.05$) correlations in the upper left panel, following correction for serial autocorrelation. Lower: area-weighted mean temperatures over Greenland (yellow box) and Fennoscandia (green box), illustrating the decadal-scale antiphasing between the regions underlying the low correlations between the region.

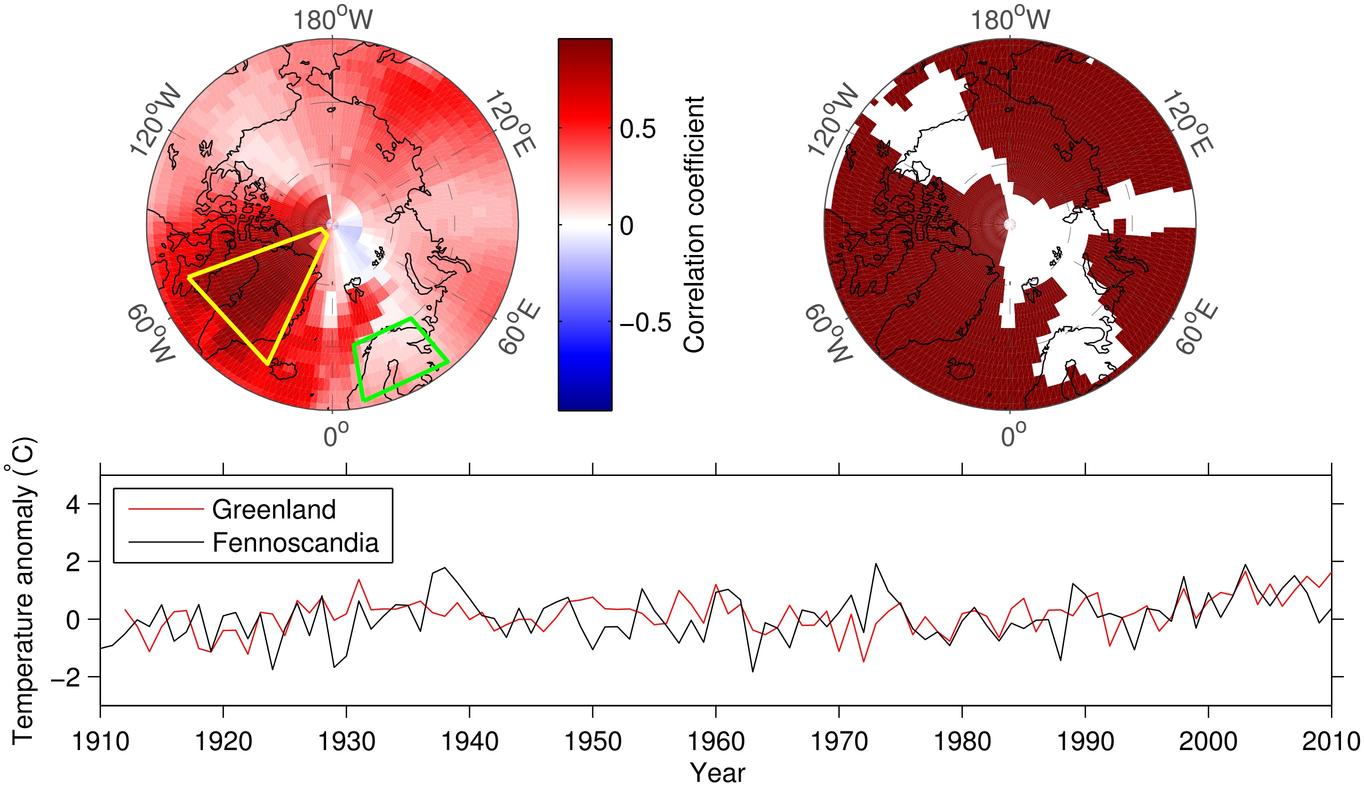


Figure S3. As in figure S2, but for June-August instead of annual temperatures.

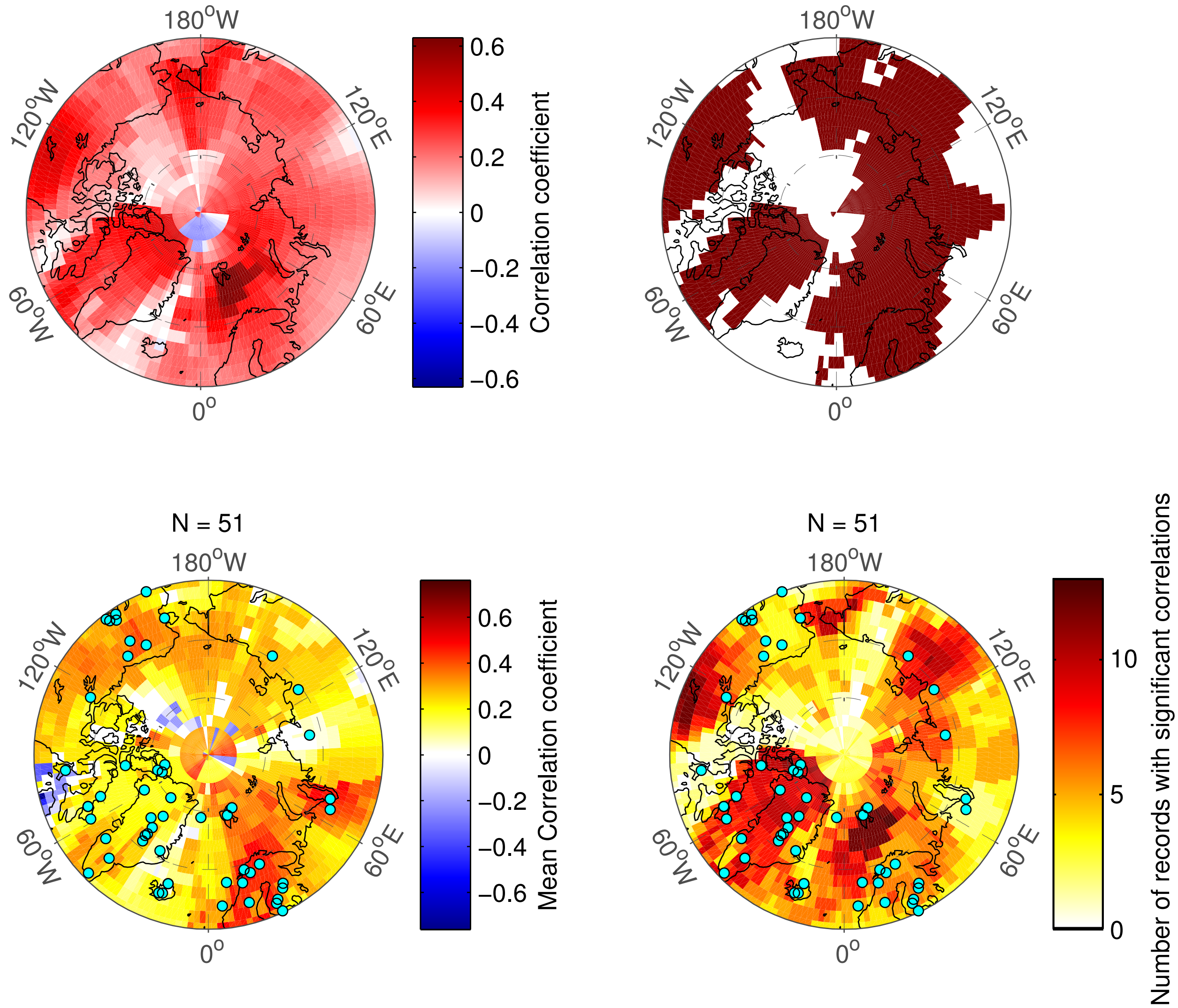


Figure S4. As in figure 3, but for June-August instead of annual temperatures.

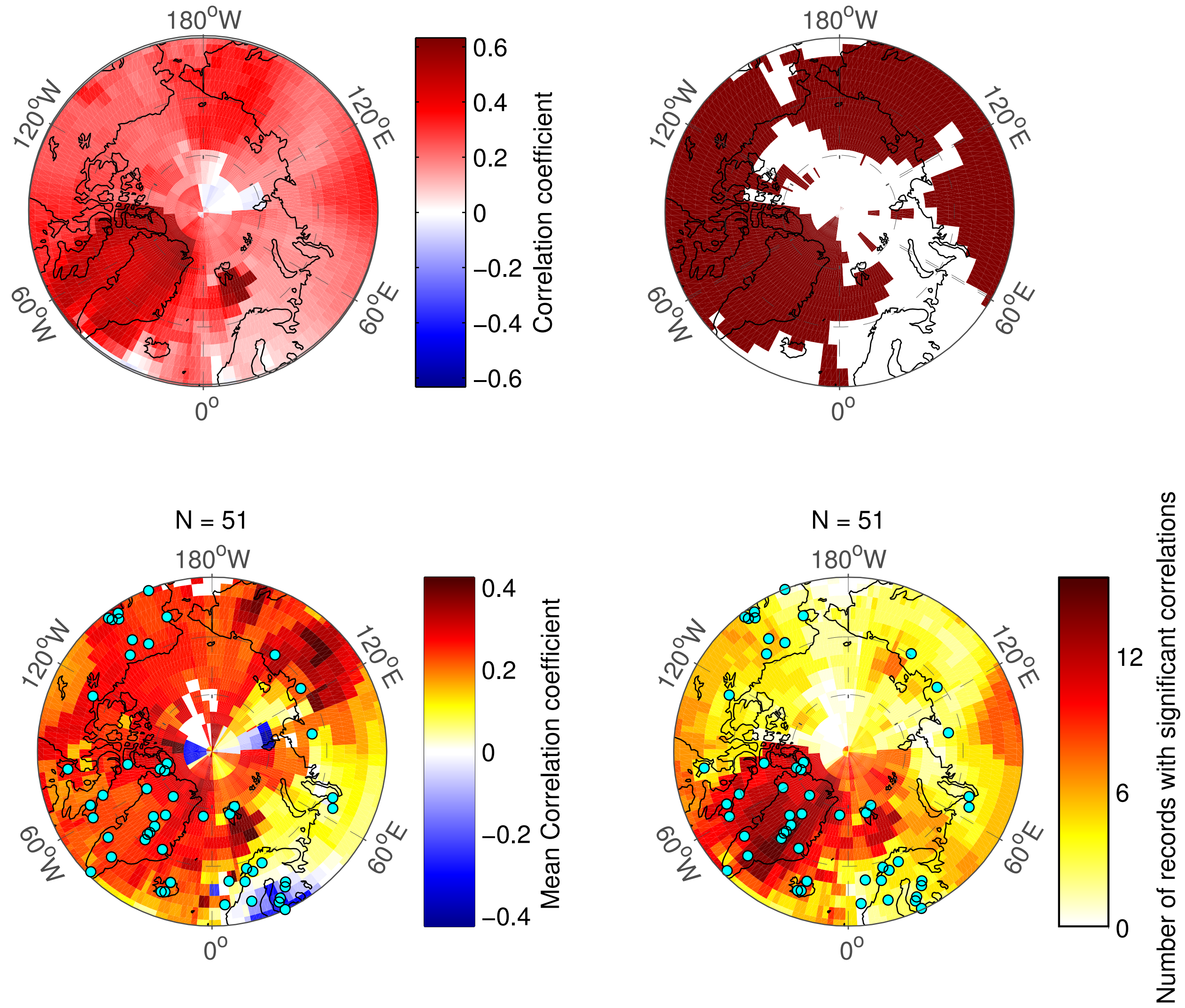


Figure S5. As in figure 3, but for October-March instead of annual temperatures.
